# Global, regional, and national cardiovascular disease burden attributable to smoking from 1990 to 2021: Findings from the GBD 2021 Study

**DOI:** 10.18332/tid/200072

**Published:** 2025-01-31

**Authors:** Shuaijie Zhu, Jian Gao, Liangliang Zhang, Wanguo Dong, Wei Shi, Heng Guo, Xiaoyu Zhang, Tianfeng Hua, Min Yang

**Affiliations:** 1 Laboratory of Cardiopulmonary Resuscitation and Critical Illness, The Second Affiliated Hospital of Anhui Medical University, Hefei, People’s Republic of China; 2 The Second Department of Critical Care Medicine, The Second Affiliated Hospital of Anhui Medical University, Hefei, People’s Republic of China

**Keywords:** cardiovascular disease, smoking, years lived with disability, estimated annual percentage change

## Abstract

**INTRODUCTION:**

Smoking poses a serious threat to cardiovascular health. The aim of the study is to evaluate the global, regional, and national burden of smoking-attributable cardiovascular disease (CVD) and set priorities for future tobacco control.

**METHODS:**

Data on deaths and years lived with disability (YLDs) for smoking-related CVD from 1990 to 2021, including age-standardized rates (ASRs), were sourced from the Global Burden of Disease (GBD) 2021. This study is a secondary descriptive analysis of the GBD 2021 data. We used the estimated annual percentage change (EAPC) to reflect temporal trends in disease burden and conducted a sensitivity analysis using the average annual percentage change (AAPC) to corroborate the findings. We also analyzed the relationship between disease burden and the Sociodemographic Index (SDI).

**RESULTS:**

In 2021, smoking-related CVD caused 2.25 million deaths and 3.09 million YLDs globally, marking increases of 26.16% and 59.73% from 1990, respectively. However, from 1990 to 2021, the global age-standardized mortality rate (ASMR) and age-standardized years lived with disability rate (ASYR) for smoking-related CVDs decreased, with EAPCs of -1.94 and -0.92, respectively. Between 1990 and 2021, Australasia and Tropical Latin America experienced the largest declines in ASMR and ASYR, with EAPCs of -5.54 and -2.63, respectively, while Lesotho and Mali had the largest increases, with EAPCs of 2.68 and 1.67, respectively. Throughout the period, the burden of smoking-related CVD was consistently higher in men. Moreover, the trend of decline in disease burden was slower in men compared to women (EAPC for ASMR: -1.78 for men vs -3.25 for women). In 2021, countries with higher SDI also had higher ASYR.

**CONCLUSIONS:**

Although the global burden of smoking-related CVD has declined over the past three decades, the challenge remains severe, particularly in less developed countries and regions. More proactive and effective tobacco control measures should be urgently implemented in countries where the burden of smoking-related CVD is severe.

## INTRODUCTION

Cardiovascular disease (CVD) encompasses a range of disorders characterized by heart and blood vessel dysfunction, including heart disease, stroke, aneurysms, and peripheral vascular diseases^1^. Due to its sudden onset and high disability rate, CVD has become the leading cause of death worldwide^2^. The prevalence of this disease continues to rise globally, affecting both developed and developing countries. According to a report by the World Health Organization, approximately 17.9 million people died from CVD in 2019, accounting for 32% of global deaths. These fatalities are predominantly concentrated in underdeveloped regions^3^. Numerous factors have been identified as closely associated with the onset and progression of CVD, including genetics, dietary habits, obesity, smoking, excessive alcohol consumption, and socioeconomic factors^4-6^. Importantly, most risk factors associated with CVD are preventable, offering new avenues for the prevention of this disease.

Smoking is a common risk factor for various diseases, including cancer, CVD, and respiratory diseases^7-9^. A recently published global study on disease risk factors identified smoking as a major health threat worldwide, ranking just behind particulate matter pollution and high systolic blood pressure^10^. Given the substantial health burden caused by smoking and its close association with various diseases, accurately assessing the impact of smoking on the burden of different diseases is of great significance for guiding the formulation of future public health policies. However, existing research on smoking-related diseases has primarily focused on the impact of smoking on cancer, while studies on its contribution to CVD, the leading cause of death worldwide, remain limited. Specifically, comprehensive analyses of the temporal and spatial trends in the burden of CVD attributable to smoking are notably lacking.

The GBD 2021 study incorporated a wide range of original research and employed rigorous methodologies to assess the health burden of 88 risk factors and 371 diseases and injuries across 21 GBD regions and 204 countries from 1990 to 2021^10,11^. The results were systematically presented by age, gender, region, and year. This study aims to use GBD 2021 to comprehensively analyze the spatial and temporal trends of cardiovascular diseases attributable to smoking, and to explore the relationship between the burden of these diseases and the level of social development. In addition, the trends of subtypes of cardiovascular diseases are also analyzed in this study.

## METHODS

### Data sources

This is a secondary dataset analysis using data on the burden of CVD attributed to smoking. The data on the burden of CVD attributable to smoking used in this study were obtained from the publicly accessible and free database of the GBD 2021, which requires no special permissions. We obtained the relevant data from 1990 to 2021 for global, 21 GBD regions, and 204 countries and territories, across different genders and age groups for analysis. Given the overlap between mortality and disability-adjusted life years (DALYs) – which represent the sum of years of life lost due to premature mortality and years lived with disability – in measuring the burden of disease, this study uses crude mortality numbers, years lived with disability (YLDs), which reflect the years spent with any long-term or short-term disability caused by these diseases, and corresponding age-standardized rates to illustrate the disease burden^12^. The GBD 2021 dataset provides global estimates of health burdens, including mortality, morbidity, and other related factors, which are derived from large-scale data collections analyzed using advanced modeling techniques. Therefore, the figures should be interpreted as approximations based on the available data^13^.

The Sociodemographic Index (SDI) is a composite indicator that reflects the social and economic development levels influencing health outcomes across different regions. It is derived by calculating the geometric mean of three parameters: mean years of education for individuals aged ≥15 years, fertility rates for women aged <25 years, and lag-distributed income per capita. SDI values range between 0 and 1^14^. Notably, when reporting SDI values in GBD 2021, the calculated SDI values were multiplied by 100, resulting in a scale from 0 (highest fertility, lowest education, and lag-distributed income) to 100 (lowest fertility, highest education, and lag-distributed income). To describe the relationship between SDI values and the disease burden across different countries and regions, all areas were divided into five SDI categories: low (<0.47), low-middle (0.62–0.71), high-middle (0.71–0.81), and high (>0.81)^11^.

### Definition of concepts

The definition of cardiovascular disease in the GBD 2021 study aligns with the definition used in the original study. Heart and vascular diseases, such as ischemic heart disease, aortic aneurysm, and peripheral artery disease, share similar risk factors and are closely interconnected. For statistical analysis purposes, these conditions are grouped under the category of cardiovascular disease in the GBD 2021 study. The definition of smoking in GBD 2021 is the current use of any tobacco products or former smokers who have quit all tobacco products for at least six months^10^.

### Estimation framework

The GBD 2021 study uses the Comparative Risk Assessment (CRA) framework to assess the health burden attributable to risk factors. Briefly, this process is divided into the following seven steps: 1) quantifying the relative risk of health outcomes associated with risk factors; 2) collecting exposure levels and population distributions for each risk factor; 3) determining the theoretical minimum risk exposure level; 4) estimating the population attributable fraction (PAF); 5) calculating age-specific total exposure values; 6) adjusting the PAF; and 7) evaluating the attributable burden. Notably, GBD 2021 introduced a new method, counterfactual risk analysis, which addresses unexplained heterogeneity among different input datasets and supplements traditional relative risk (RR) result^10,15^.

### Statistical analysis

Estimated annual percentage change (EAPC) was used to assess trends in age-standardized rates over the study period (1990–2021). EAPC was calculated by fitting a linear regression model to the natural logarithm of the age-standardized rates against the calendar year, following the formula y = a + bx + E, where y represents the natural logarithm of the age-standardized rate, x represents the calendar year, E is the standard error, and b is the calculated EAPC. The sign of b indicates the direction of the trend in age-standardized rates. The 95% confidence interval (CI) for EAPC was calculated using the formula 100 [exp(b) – 1]. The trend in age-standardized rates was interpreted using EAPC and its 95% CI: if b>0 and the lower bound of the CI (LCI) >0, it indicates an increasing disease burden; if b<0 and the upper bound of the CI (UCI) <0, it indicates a decreasing disease burden; if the 95% CI includes 0, the disease burden is considered stable over the period^16^. To further corroborate the findings of the EAPC, alternative techniques such as jointpoint analysis were considered. Jointpoint analysis will be used as a sensitivity analysis for key outcomes. The results, presented as AAPC (average annual percent change), corroborate the trends observed with EAPC, reinforcing the robustness of the finding.

To provide a straightforward depiction of the status of disease burden, data from the starting point (1990) and the endpoint (2021) of the study period were selected. These two specific years illustrate the burden at the beginning and end of the study period.

The subgroup analyses conducted in this study are descriptive in nature, aiming to summarize patterns and trends in the burden of CVD attributable to smoking across different genders, age groups, countries, and regions. No hypothesis testing or statistical inference was performed, and therefore, adjustments for multiple comparisons were not applied.

To evaluate the relationship between the SDI and the associated disease burden, Spearman regression analysis was employed. This non-parametric method is particularly suitable for assessing monotonic relationships between variables that do not meet the normality assumption. The results are reported as Spearman’s correlation coefficient (ρ) and the corresponding p-value, which indicates the statistical significance of the observed correlation^17^. A p<0.05 was considered statistically significant.

All data analysis and visualization were performed using R software, with results rounded to two decimal places. The results, including correlation coefficients and statistical outputs, were rounded to two decimal places for clarity.

## RESULTS

### Global analysis of smoking-induced CVD burden and evolving trends

The global number of deaths from CVD attributable to smoking increased from 1781364 in 1990 to 2247325 in 2021, representing a 26.16% rise. However, the ASMR declined from 46.47 per 100000 in 1990 to 26.29 per 100000 in 2021, with an EAPC of -1.94 (95% CI: -2.02 – -1.87) and a sensitivity analysis AAPC of -1.85 (95% CI: -2.06 – -1.63) (Table 1). Meanwhile, the YLDs due to smoking-related CVD increased from 1936297 in 1990 to 3092855 in 2021, marking a 59.73% rise (Supplementary file Table 1). Correspondingly, the ASYR decreased from 46.59 per 100000 to 35.49 per 100000, with an EAPC of -0.92 (95% CI: -0.93 – -0.9) and a sensitivity analysis AAPC of -0.88 (95% CI: -0.89 – -0.86).

**Table 1:** Age-standardized mortality and YLDs rates across GBD regions and global in 1990 and 2021, and their temporal trends from 1990 to 2021 (based on GBD 2021 study data)

*Location*	*Age-standardized mortality rate per 100000 population*				*Age-standardized YLDs rate per 100000 population*			
	*1990*	*2021*	*EAPC (95% CI)*	*AAPC (95% CI)*	*1990*	*2021*	*EAPC (95% CI)*	*AAPC (95% CI)*
Global	46.47	26.29	-1.94 (-2.02 – -1.87)	-1.85 (-2.06 – -1.63)	46.59	35.49	-0.92 (-0.93 – -0.9)	-0.88 (-0.89 – -0.86)
Andean Latin America	13.52	6.77	-2.44 (-2.8 – -2.09)	-2.2 (-2.63 – -1.77)	13.54	11.46	-0.63 (-0.73 – -0.53)	-0.55 (-0.59 – -0.5)
Australasia	35.41	6.64	-5.54 (-5.68 – -5.4)	-5.28 (-5.5 – -5.06)	37.98	20.19	-2.19 (-2.24 – -2.13)	-2.04 (-2.08 – -2.01)
Caribbean	39.24	20.36	-2.23 (-2.36 – -2.1)	-2.02 (-2.35 – -1.7)	29.08	20.83	-1.23 (-1.33 – -1.14)	-1.07 (-1.09 – -1.04)
Central Asia	54.73	41.83	-1.16 (-1.57 – -0.75)	-0.85 (-1.03 – -0.67)	45.56	40.41	-0.34 (-0.48 – -0.2)	-0.37 (-0.43 – -0.3)
Central Europe	75.72	28.05	-3.59 (-3.73 – -3.45)	-3.16 (-3.39 – -2.93)	72.13	48.38	-1.4 (-1.44 – -1.35)	-1.28 (-1.32 – -1.24)
Central Latin America	22.70	10.72	-2.8 (-2.98 – -2.63)	-2.43 (-2.56 – -2.3)	21.94	11.68	-2.24 (-2.33 – -2.14)	-2 (-2.08 – -1.92)
Central Sub-Saharan Africa	16.69	12.26	-1.06 (-1.33 – -0.8)	-0.97 (-1.07 – -0.87)	13.51	10.85	-0.64 (-0.8 – -0.48)	-0.7 (-0.73 – -0.68)
East Asia	56.17	38.99	-0.98 (-1.15 – -0.8)	-1.23 (-1.44 – -1.03)	56.67	54.98	0.05 (-0.05 – 0.15)	-0.13 (-0.23 – -0.03)
Eastern Europe	61.09	48.62	-1.35 (-2.07 – -0.64)	-0.71 (-1.58–0.16)	48.60	47.94	-0.03 (-0.22 – 0.17)	-0.03 (-0.1– 0.05)
Eastern Sub-Saharan Africa	18.22	11.90	-1.63 (-1.74 – -1.52)	-1.35 (-1.42 – -1.27)	19.74	14.66	-1.06 (-1.13 – -0.98)	-0.95 (-0.99 – -0.91)
High-income Asia Pacific	27.56	8.24	-4.08 (-4.17 – -3.99)	-3.82 (-3.94 – -3.71)	62.51	34.25	-2.21 (-2.31 – -2.12)	-1.93 (-1.99 – -1.87)
High-income North America	46.69	15.87	-3.85 (-4.05 – -3.66)	-3.46 (-3.61 – -3.3)	53.94	36.41	-1.55 (-1.63 – -1.46)	-1.29 (-1.34 – -1.24)
North Africa and Middle East	61.71	36.39	-1.86 (-1.92 – -1.81)	-1.68 (-1.78 – -1.57)	40.72	33.40	-0.75 (-0.79 – -0.7)	-0.63 (-0.66 – -0.6)
Oceania	49.98	39.34	-0.83 (-0.87 – -0.79)	-0.77 (-0.84 – -0.69)	41.73	35.94	-0.58 (-0.64 – -0.53)	-0.48 (-0.51 – -0.44)
South Asia	36.81	27.23	-0.93 (-1.02 – -0.85)	-0.93 (-1.25 – -0.6)	28.55	20.54	-1.09 (-1.12 – -1.06)	-1.06 (-1.08 – -1.04)
Southeast Asia	47.86	36.49	-0.91 (-1 – -0.82)	-0.89 (-0.98 – -0.81)	51.93	43.49	-0.64 (-0.7 – -0.58)	-0.57 (-0.59 – -0.55)
Southern Latin America	34.96	11.30	-3.49 (-3.55 – -3.44)	-3.55 (-3.92 – -3.18)	43.55	26.92	-1.75 (-1.82 – -1.68)	-1.55 (-1.58 – -1.52)
Southern Sub-Saharan Africa	26.69	16.08	-1.76 (-2.09 – -1.44)	-1.69 (-2.15 – -1.22)	41.86	20.65	-2.41 (-2.54 – -2.29)	-2.25 (-2.3 – -2.21)
Tropical Latin America	55.64	16.51	-4.06 (-4.21 – -3.9)	-3.81 (-3.99 – -3.63)	42.97	20.53	-2.63 (-2.76 – -2.51)	-2.35 (-2.41 – -2.3)
Western Europe	43.20	10.15	-4.88 (-5 – -4.76)	-4.6 (-4.69 – -4.51)	50.50	30.29	-1.75 (-1.8 – -1.71)	-1.63 (-1.66 – -1.59)
Western Sub-Saharan Africa	11.12	8.31	-1.06 (-1.23 – -0.88)	-0.93 (-1.04 – -0.82)	12.94	10.50	-0.74 (-0.85 – -0.64)	-0.67 (-0.7 – -0.65)

YLDs: years lived with disability. GBD: global burden of disease. EAPC: estimated annual percentage change. AAPC: average annual percent change.

### Regional disparities in CVD attributable to smoking

In 2021, East Asia had the highest number of deaths attributed to CVD, with 802131.94 deaths (Supplementary file Table 1). Conversely, Oceania had the lowest number of deaths, with 3456.16. Additionally, Eastern Europe and Australia had the highest and lowest ASMRs, at 48.62 and 6.64 per 100000, respectively. Similar to the absolute burden of deaths from smoking-related CVD, East Asia and Oceania also had the highest and lowest YLDs attributable to smoking-related CVD, with 1219471.56 and 3460.09 person-years, respectively After standardizing for age, East Asia exhibited the highest ASYR at 54.98 per 100000, while West Sub-Saharan Africa had the lowest rate at 10.50 per 100000.

A review of 21 GBD regions from 1990 to 2021 demonstrates a uniform decline in cardiovascular mortality linked to smoking across all regions. Australia exhibited the most notable decrease, with an EAPC of -5.54 (95% CI: -5.68 – -5.4) and a sensitivity analysis AAPC of -5.28 (95% CI: -5.5 – -5.06). However, while ASYR generally decreased across most regions, it remained stable in East Asia and Eastern Europe, with EAPCs of 0.05 (-0.05–0.15) and -0.03 (-0.22–0.17), respectively. Sensitivity analysis using AAPC showed values of -0.13 (95% CI: -0.23 – -0.03) for East Asia and -0.03 (95% CI: -0.10–0.05) for Eastern Europe. Notably, Tropical Latin America experienced the steepest reduction in YLDs burden, with an EAPC of -2.63 (95% CI: -2.76 – -2.51) (Table 1).

### Variations in the national burden of CVD attributable to smoking

At the national level, in 2021, China had the highest number of CVD deaths and YLDs attributed to smoking, with 784390.90 deaths and 1188145.93 person-years, respectively (Supplementary file: Figure 1 and Table 2). However, the countries with the highest ASYR and ASMR were Kiribati and Nauru, with 112.63 and 110.04 per 100000 people, respectively. Analysis of trends in disease burden over this period reveals that most countries saw a decline, with only a few experiencing increases or stability. Ireland saw the steepest declines in both ASMR and ASYR, with EAPCs of -6.73 (95% CI: -6.96 – -6.5) and -3.38 (95% CI: -3.54 – -3.22), respectively. Sensitivity analysis using AAPC showed values of -6.27 (95% CI: -6.66 – -5.88) for ASMR and -2.89 (95% CI: -2.96 – -2.82) for ASYR. Conversely, Lesotho recorded the fastest rise in ASMR, and Mali experienced the most rapid increase in ASYR, with corresponding EAPCs of 2.68 and 1.27, respectively. Sensitivity analysis using AAPC showed values of 1.98 (95% CI: 1.72 – 2.25) for ASMR in Lesotho and 1.01 (95% CI: 0.96 – 1.06) for ASYR in Mali. (Supplementary file: Figure 2 and Table 3).

### Disease burden differences by gender and age

**Figure 1:**
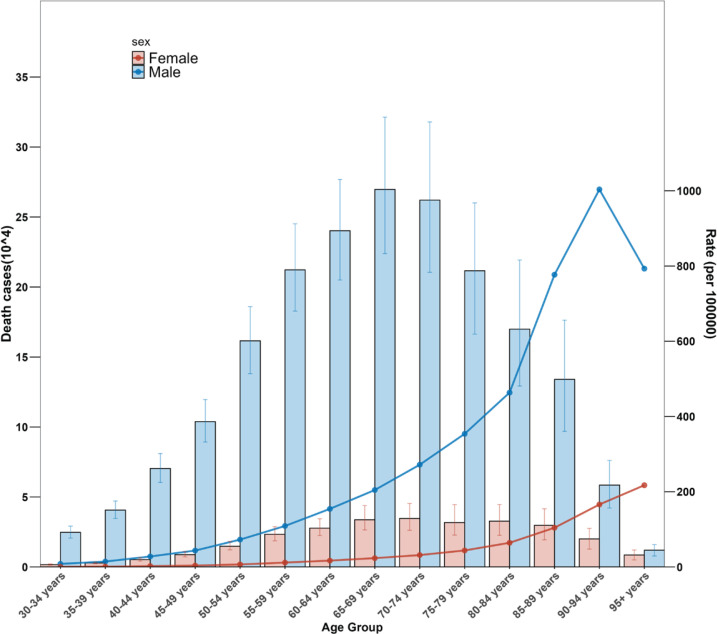
Global age patterns of cardiovascular disease burden attributable to smoking by gender in 2021: Death cases and mortality rates

**Figure 2:**
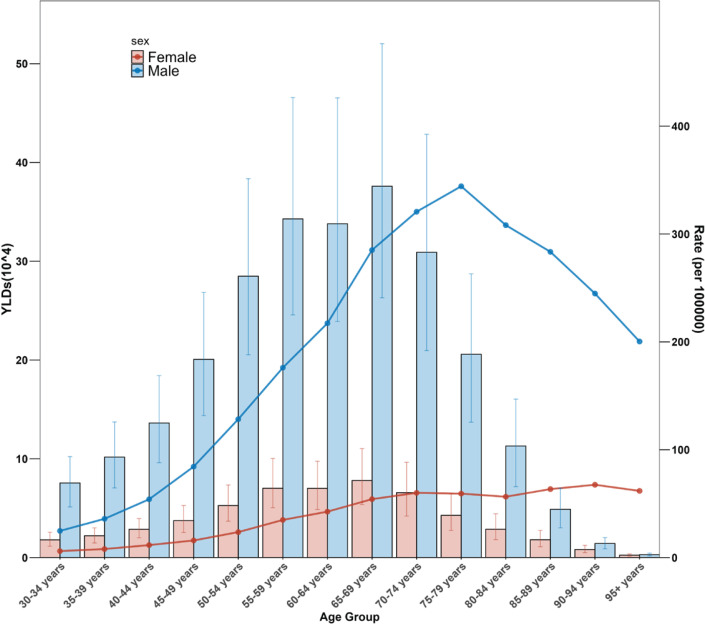
Global age patterns of cardiovascular disease burden attributable to smoking by gender in 2021: YLDs and YLD rates

In 2021, globally, deaths and YLDs due to CVD attributable to smoking were higher in men than in women (Table 2). Analysis of age groups revealed that the highest mortality burden for men was in the age group of 65–69 years, while for women, it was in the 70–74 years age group (Figure 1). Similarly, the highest burden of YLDs was found in men aged 55–59 years and in women aged 65–69 years (Figure 2). Analysis of temporal trends showed that while the disease burden declined for both genders, the rates of decline varied. The EAPCs in the ASMR and ASYR were -1.78 and -0.75 for men, respectively, compared to -3.25 and -1.76 for women (Table 3).

**Table 2:** Number of cardiovascular disease mortality and YLDs attributable to smoking by gender and SDI regions globally in 1990 and 2021, and the percent change from 1990 to 2021 (based on GBD 2021 study data)

	*Mortality cases*			*YLDs*		
	*1990*	*2021*	*Percent change*	*1990*	*2021*	*Percent change*
**Gender**						
Male	1470868.30	1971508.14	34.04	1503802.57	2549934.91	69.57
Female	310496.09	275817.24	-11.17	432494.07	542920.43	25.53
**SDI regions**						
High SDI	493474.08	278893.18	-43.48	600344.85	646019.18	7.61
High-middle SDI	526805.73	640947.02	21.67	568336.61	926399.08	63.00
Middle SDI	466238.01	802796.41	72.19	498286.62	1033278.35	107.37
Low-middle SDI	239254.15	434791.55	81.73	215783.22	395522.77	83.30
Low SDI	52927.22	87895.46	66.07	50957.95	88906.28	74.47

YLDs: years lived with disability. SDI: sociodemographic index. GBD: global burden of disease.

**Table 3:** Age-standardized mortality and YLDs rates of cardiovascular disease attributable to smoking by gender and SDI regions globally in 1990 and 2021, and time trends from 1990 to 2021 (based on GBD 2021 study data)

	*Age-standardized mortality rate* *per 100000 population*			*Age-standardized YLDs rate* *per 100000 population*		
	*1990*	*2021*	*EAPC (95% CI)*	*1990*	*2021*	*EAPC (95% CI)*
**Gender**						
Male	85.43	50.50	-1.78 (-1.85 – -1.71)	76.92	61.33	-0.75 (-0.77 – -0.72)
Female	15.39	5.94	-3.25 (-3.35 – -3.14)	19.95	11.96	-1.76 (-1.8 – -1.72)
**SDI regions**						
High SDI	44.91	13.51	-4.12 (-4.26 – -3.98)	56.13	35.60	-1.66 (-1.72 – -1.6)
High-middle SDI	54.49	32.72	-1.9 (-2.19 – -1.6)	54.97	47.77	-0.44 (-0.47 – -0.41)
Middle SDI	47.49	31.03	-1.35 (-1.41 – -1.28)	43.91	36.96	-0.51 (-0.56 – -0.47)
Low-middle SDI	39.58	30.71	-0.75 (-0.8 – -0.7)	32.31	25.73	-0.75 (-0.77 – -0.73)
Low SDI	23.72	17.72	-0.98 (-1.07 – -0.9)	20.20	15.33	-0.98 (-1.02 – -0.94)

YLDs: years lived with disability. SDI: sociodemographic Index. EAPC: estimated annual percentage change. GBD: global burden of disease.

### SDI and its role in shaping disease burden

In 2021, the mortality and YLDs attributed to CVD due to smoking were lower in low-SDI regions compared to other regions (Table 2). An analysis of disease burden trends across the quintile SDI regions revealed a downward trend in all quintiles, with the most significant decline observed in the high SDI quintile. The EAPCs for ASMR and ASYR in the high SDI level were -4.12 and -1.66, respectively (Table 3).

**Figure 3:**
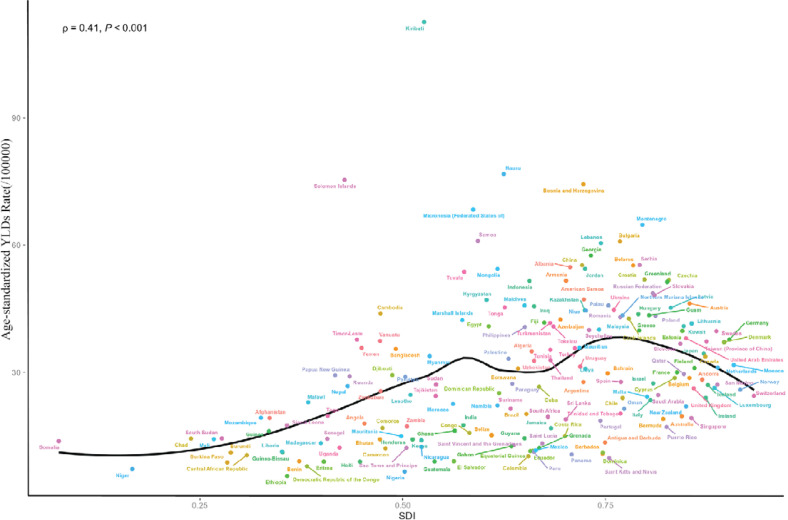
The correlation between age-standardized YLDs rates and SDI across 204 countries in 2021

**Figure 4:**
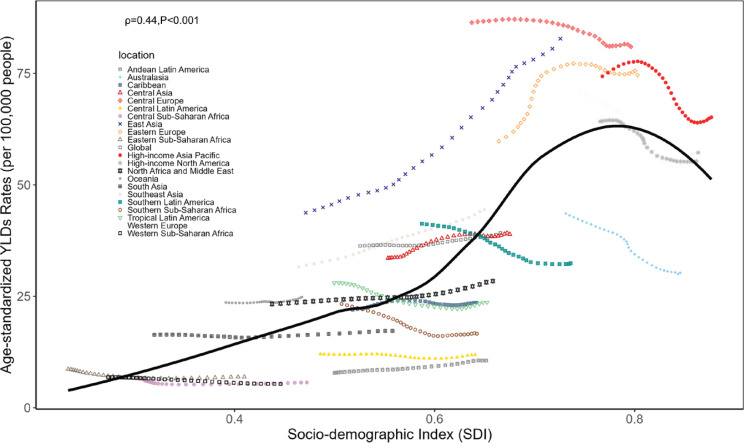
The correlation between age-standardized YLDs rates and SDI in global and 21 GBD regions from 1990 to 2021

At the national level in 2021, we observed a positive correlation between ASYR and SDI in smoking-related CVD burden. This non-linear fit indicates that countries with higher SDI levels have a greater ASYR CVD burden compared to those with lower SDI levels (Figure 3). To analyze the relationship between global and GBD regional ASMR, ASYR, and SDI, we integrated the relevant data. Our analysis revealed that, apart from regions such as Western Europe, Central Asia, and East Asia – where the disease burden initially rose with increasing SDI and then declined – other regions experienced a decrease in smoking-related CVD as SDI increased (Figure 4).

### Burden of disease attributable to smoking cardiovascular disease subtypes

Stroke and ischemic heart disease are the primary subcategories of CVDs attributable to smoking. In 2021, the ASMR and ASYR for smoking-attributable strokes were 9.69 and 23.27 per 100000, respectively. Similarly, the ASMR and ASYR for smoking-attributable ischemic heart disease were 15.76 and 8.33 per 100000, respectively. A temporal trend analysis of CVD subtypes indicates that lower extremity peripheral arterial disease experienced the most significant decline, with EAPCs in ASMR and ASYR calculated at -2.63 and -1.59, respectively (Table 4).

**Table 4:** Age-standardized mortality and YLDs rates of cardiovascular disease subtypes attributable to smoking in 1990 and 2021, and time trends from 1990 to 2021 (based on GBD 2021 study data)

	*Age-standardized mortality rate* *per 100000 population*			*Age-standardized mortality rate* *per 100000 population*		
	*1990*	*2021*	*EAPC (95% CI)*	*1990*	*2021*	*EAPC (95% CI)*
Atrial fibrillation and flutter	0.15	0.12	-0.77 (-0.83 – -0.71)	3.27	2.64	-0.69 (-0.72 – -0.66)
Stroke	18.08	9.69	-2.15 (-2.24 – -2.05)	30.74	23.27	-0.94 (-0.96 – -0.93)
Lower extremity peripheral arterial disease	0.33	0.16	-2.63 (-2.83 – -2.44)	2.02	1.26	-1.59 (-1.63 – -1.55)
Ischemic heart disease	26.93	15.76	-1.81 (-1.86 – -1.75)	10.57	8.33	-0.8 (-0.83 – -0.77)
Aortic aneurysm	0.98	0.56	-2.17 (-2.3 – -2.05)			

YLDs: years lived with disability. EAPC: estimated annual percentage change. GBD: global burden of disease.

## DISCUSSION

This study provides a comprehensive descriptive analysis of the burden of CVD attributable to smoking at the global, regional, and national levels from 1990 to 2021. Notably, although the ASRs of death and YLDs due to smoking-related CVD have decreased globally and across GBD regions, the overall burden has continued to increase. In contrast to the global and GBD regional trends, the temporal trends in disease burden across the 204 countries and regions analyzed in the GBD 2021 study were not uniformly decreasing; in some countries and regions, the burden remained stable or even increased. A thorough analysis of the current state and evolving trends of smoking-attributable CVD is essential for understanding the dynamic nature of this global public health challenge and for guiding the development of effective prevention strategies.

In 2021, China had the highest number of deaths and YLDs due to smoking-related CVD, while Kiribati and Nauru had the highest ASYR and ASMR. EAPC shows that Ireland experienced the most significant decrease in death and disability burdens, while Lesotho and Mali exhibited the most notable increases in ASMR and ASYR, respectively. The uneven distribution and varying trends in disease burden across countries and regions are influenced by local demographic factors and smoking prevalence. A study on tobacco use in China shows that while smoking prevalence has decreased over the past few decades, the country still accounts for one-third of the world’s tobacco consumption each year, resulting in 1 million smoking-related deaths annually^18,19^. Similarly, Nauru and Kiribati face a significant disease burden due to their high smoking rates^20^, while the heavy disease burden in China is primarily driven by its large population. According to a report by Irish health authorities, smoking rates in Ireland dropped from 27% in 2004 to 18% in 2023, leading to a significant reduction in the associated disease burden^21^. In contrast, the decline in smoking rates in Lesotho and Mali has been minimal in recent years, with male smoking rates in Lesotho even increasing, contributing to the slow decline or even rise in the CVD burden attributed to smoking in these regions^22^. These findings suggest that developing and implementing targeted anti-smoking goals tailored to the specific needs of different regions is a crucial step in reducing the burden of smoking-related CVD in these areas. For instance, the Chinese government is currently implementing the Healthy China 2030 strategy, which aims to reduce the smoking rate to below 20% by 2030, with the aim of effectively lowering the burden of smoking-related diseases in the country^23^.

In 2021, the burden of CVD attributable to smoking, including both mortality and YLDs, was higher in men than in women. Moreover, the peak in mortality and YLDs occurred earlier in men than in women. The gender differences in disease burden may not only be associated with smoking prevalence but also with the age of smoking initiation and hormonal differences. A risk analysis of smoking initiation age found that each year of delay in starting smoking reduces the risk of cardiovascular events by 4%. Additionally, men generally start smoking at an earlier age than women^24,25^. Besides the age of smoking initiation, hormones also play a critical role in regulating cardiovascular function. For instance, estrogen in the endocrine system can reduce the incidence of cardiovascular events by improving vasodilation, regulating lipid metabolism, and exerting anti-atherosclerotic effects^26^. These factors are crucial in explaining the gender differences in the distribution of smoking-attributable cardiovascular disease.

From 1990 to 2021, in most GBD regions, the burden of CVD attributable to smoking gradually decreased with the rise in the SDI. As socioeconomic levels improved, public awareness of the harms of smoking increased, leading more individuals to take measures such as quitting smoking, which effectively reduced smoking-related health risks. Furthermore, our study revealed a nonlinear positive correlation at the national level in 2021 between the SDI and the ASYR due to smoking-related CVD, suggesting that higher SDI countries tend to have a greater YLDs burden. In low-SDI regions, the burden of CVD due to smoking may be partially driven by deficiencies in the local healthcare system^27^. In contrast, in high-SDI regions, the increase in age-standardized YLDs rates of cardiovascular diseases may be more closely associated with other cardiovascular risk factors in these regions, such as obesity, stress, and longer life expectancy^28,29^. A global study on obesity revealed that the prevalence of obesity is higher in developed countries than in developing nations^30^. Notably, Kiribati, despite being a low-SDI region, had an ASYR significantly above the average, likely due to high smoking rates driven by lax tobacco control measures. These findings suggest that tobacco-related CVD prevention strategies should be tailored to the specific needs of regions with different SDI levels.

### Strengths and limitations

This study offers valuable contributions, including a novel assessment of the global, regional, and national burden of cardiovascular diseases attributable to smoking, providing important insights for setting global tobacco control priorities.

However, several limitations should be noted. First, the quality of the original studies included in the GBD data varies significantly, leading to considerable heterogeneity. Therefore, the estimated values derived from these studies still require validation through large cohort studies. Second, while this study uses the EAPC to capture the trends in disease burden, EAPC estimates are based on long-term high-quality data. In less developed regions, however, data quality and availability may be insufficient, which could lead to inaccurate EAPC estimates. Third, the regression analysis in this study relies on Spearman’s rank correlation, which can assess the relationship between variables but cannot establish causality. Therefore, while correlations are identified, causal inferences cannot be made. Fourth, although several known confounders, such as age and sex, were controlled for in the analysis, there may still be uncontrolled residual confounding from factors like diet and physical activity. These factors, which were not possible to account for in the current study, may introduce bias into the burden estimates. Lastly, the potential harm of emerging tobacco products, such as e-cigarettes, on cardiovascular health should not be overlooked^31^. Since the GBD study did not include these new tobacco products, future research is needed to assess their impact on cardiovascular disease.

## CONCLUSIONS

Although the global burden of CVD due to smoking has decreased over the past three decades, the overall health burden worldwide remains concerning. This is particularly evident in some developing countries, where more targeted tobacco control measures are necessary to further reduce the impact of smoking on cardiovascular disease.

## Supplementary Material



## Data Availability

The data supporting this research are available from the following source: https://vizhub.healthdata.org/gbd-results/
