# Supplementary material for: Global, regional, and national cardiovascular disease burden attributable to smoking from 1990 to 2021: Findings from the GBD 2021 Study
Source: Tob Induc Dis. 2025 Jan 31;23:10.18332/tid/200072. doi: 10.18332/tid/200072 (PMC11784507; doi:10.18332/tid/200072)
Supplement: Supplementary file 1 [file TID-23-11-s1.pdf]

Supplement to: **Global, regional, and national cardiovascular disease burden attributable to smoking from 1990 to 2021: findings from the GBD 2021 Study**

Supplementary Figures

Supplementary Figure 1: Distribution of the estimated burden of cardiovascular disease attributable to smoking in 2021 across 204 countries and territories: A) Estimated number of death cases attributable to smoking-related cardiovascular disease; B) Estimated years lived with disability (YLDs) attributable to smoking-related cardiovascular disease.

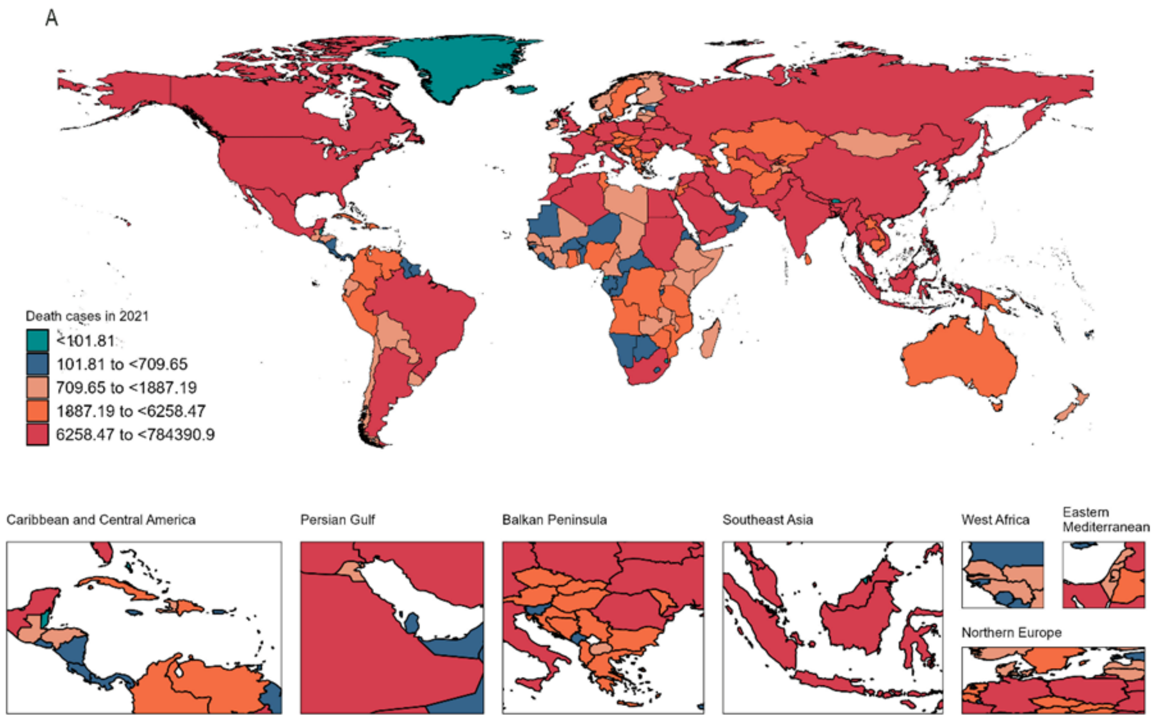

B

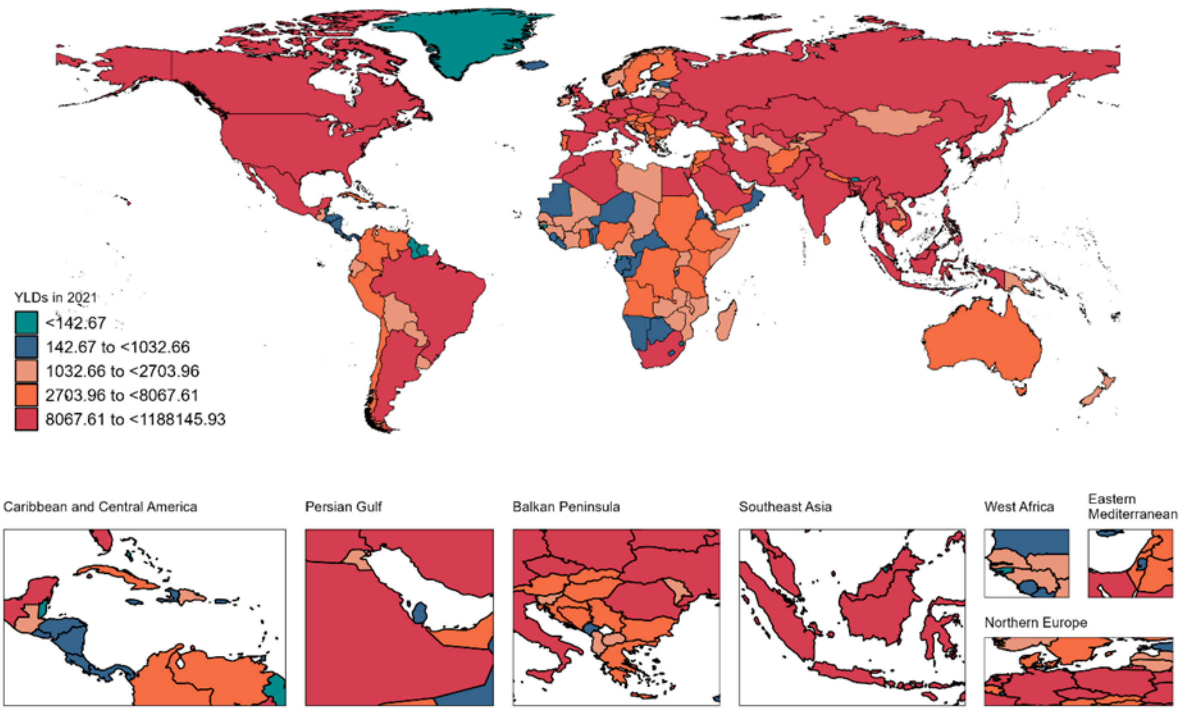

YLDs in 2021
<142.67
142.67 to <1032.66
1032.66 to <2703.96
2703.96 to <8067.61
8067.61 to <1188145.93
Caribbean and Central America
Persian Gulf
Balkan Peninsula
Southeast Asia
West Africa
Eastern Mediterranean
Northern Europe

Supplementary Figure 2: Trends in cardiovascular disease attributable to smoking across 204 countries and territories from 1990 to 2021.A: EAPC of ASMR for cardiovascular disease attributable to smoking from 1990 to 2021; B: EAPC of ASYR for cardiovascular disease attributable to smoking from 1990 to 2021. EAPC: estimated annual percentage change; ASMR: age-standardized mortality rate; ASYR: age-standardized YLDs rate.

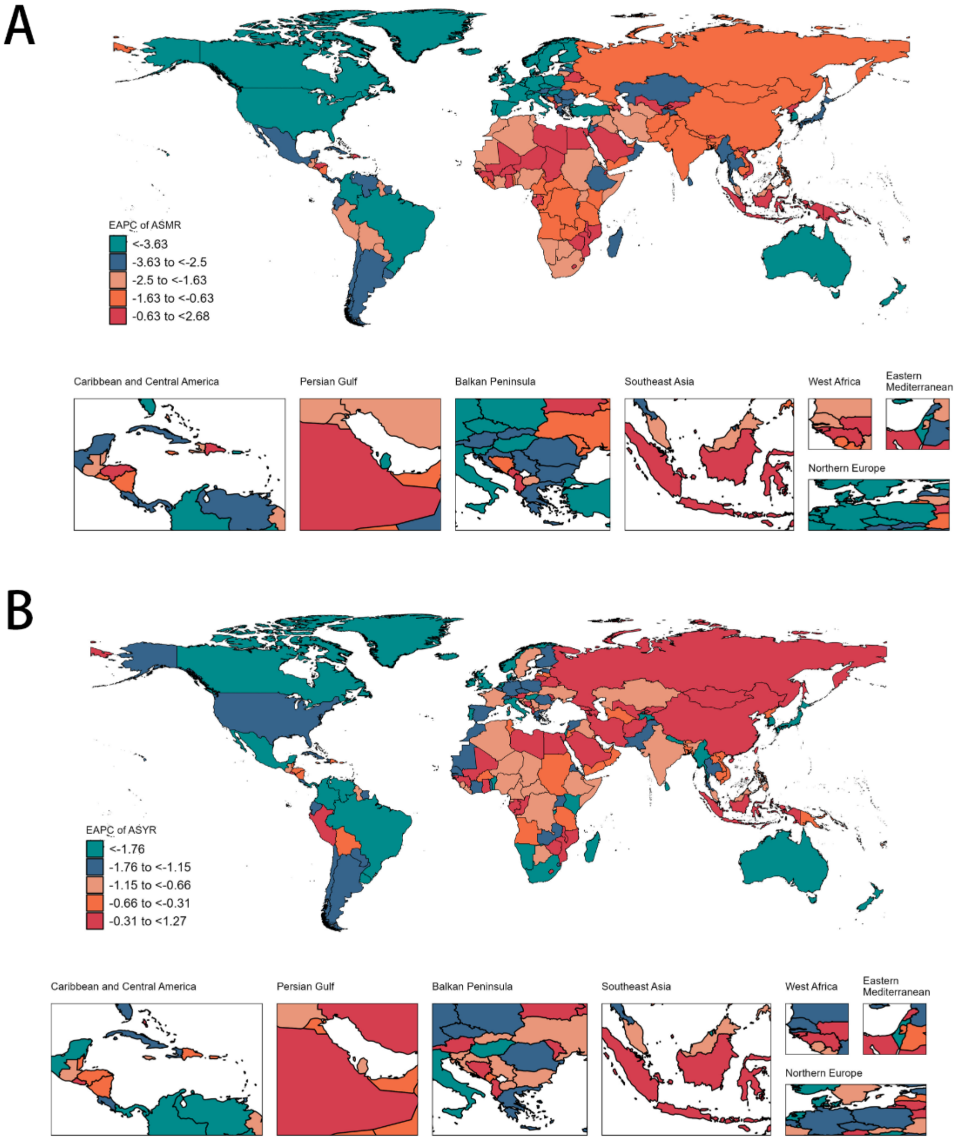

Supplementary Tables

Supplementary Table 1: Global and GBD region-specific number of mortality and YLDs attributable to smoking-related cardiovascular disease in 1990 and 2021, along with percentage changes from 1990 to 2021(based on GBD2021 study data)

location	Mortality cases			YLDs		
	1990	2021	Percentage change (%)	1990	2021	Percentage change (%)
Global	1781364.39	2247325.39	26.16	1936296.64	3092855.33	59.73
Andean Latin America	2745.35	4002.96	45.81	2977.25	7000.86	135.15
Australasia	8327.43	3541.87	-57.47	8832.37	9376.06	6.16
Caribbean	9952.81	11011.56	10.64	7760.00	11189.49	44.19
Central Asia	25854.04	33426.74	29.29	23238.57	37283.76	60.44
Central Europe	110943.18	60684.39	-45.30	108093.87	93345.00	-13.64
Central Latin America	18182.68	26814.24	47.47	19827.76	30019.48	51.40
Central Sub-Saharan Africa	3905.34	7261.70	85.94	3665.71	7751.26	111.45
East Asia	437422.50	802131.94	83.38	532647.53	1219471.56	128.95
Eastern Europe	167650.68	167160.92	-0.29	135406.10	154266.37	13.93
Eastern Sub-Saharan Africa	13236.20	20247.49	52.97	16531.52	29444.61	78.11
High-income Asia Pacific	53371.59	37584.33	-29.58	128914.01	120848.47	-6.26
High-income North America	162831.60	101909.33	-37.41	180696.71	211883.65	17.26
North Africa and Middle East	101593.04	162721.07	60.17	78947.06	176979.81	124.18
Oceania	1667.08	3456.16	107.32	1547.16	3460.09	123.64
South Asia	210848.63	390750.21	85.32	178990.76	318210.65	77.78
Southeast Asia	119189.06	239126.73	100.63	147078.21	310350.34	111.01
Southern Latin America	16196.19	9608.94	-40.67	20446.05	21936.22	7.29
Southern Sub-Saharan Africa	7203.68	9470.89	31.47	12208.57	13331.67	9.20
Tropical Latin America	50721.87	42696.29	-15.82	42732.69	53805.98	25.91
Western Europe	249891.56	97012.50	-61.18	272537.37	236887.27	-13.08
Western Sub-Saharan Africa	9629.87	16705.15	73.47	13217.37	26012.72	96.81

Abbreviations: GBD, Global Burden of Disease; YLDs, years lived with disability

Supplementary Table 2: Number of cardiovascular disease mortality and YLDs attributable to smoking in 1990 and 2021 across 204 countries and the percentage change from 1990 to 2021(based on GBD2021 study data)

	Mortality cases			YLDs		
location	1990	2021	percent change (%)	1990	2021	percent change (%)
Afghanistan	2957.93	3806.42	28.68	1155.13	2765.89	139.44
Albania	1263.01	2275.29	80.15	1303.24	2295.60	76.15
Algeria	6528.03	10471.16	60.40	5824.80	13503.19	131.82
American Samoa	11.93	21.20	77.66	15.89	24.91	56.71
Andorra	12.61	14.09	11.67	24.17	41.71	72.53
Angola	1085.03	2459.75	126.70	1062.63	2718.23	155.80
Antigua and Barbuda	10.62	10.44	-1.76	7.92	15.32	93.42
Argentina	12659.01	7032.99	-44.44	14764.57	14722.97	-0.28
Armenia	1797.89	2272.98	26.42	1570.81	2201.76	40.17
Australia	6573.83	2578.54	-60.78	7377.19	7671.79	3.99
Austria	3827.31	2314.04	-39.54	4564.19	7211.92	58.01
Azerbaijan	3544.90	5613.74	58.36	2242.12	4798.73	114.03
Bahamas	32.30	46.92	45.26	24.53	58.67	139.21
Bahrain	124.00	207.08	67.00	104.69	417.82	299.10
Bangladesh	25306.54	49008.00	93.66	20693.00	51712.13	149.90
Barbados	48.16	38.58	-19.91	39.56	52.97	33.90
Belarus	8592.37	11347.16	32.06	7715.24	8347.81	8.20
Belgium	6449.78	2288.54	-64.52	6579.60	5682.22	-13.64
Belize	18.31	31.43	71.64	17.36	49.83	187.13
Benin	248.44	349.75	40.78	346.57	582.83	68.17
Bermuda	21.94	15.49	-29.39	13.49	22.92	69.97
Bhutan	42.32	85.94	103.07	46.20	91.95	99.02
Bolivia (Plurinational State of)	665.78	860.87	29.30	502.46	1062.22	111.41
Bosnia and Herzegovina	2333.84	2426.15	3.96	3326.92	4105.72	23.41
Botswana	211.05	291.38	38.06	248.46	523.67	110.76
Brazil	49776.15	41295.72	-17.04	41776.11	52163.24	24.86
Brunei Darussalam	58.40	70.24	20.28	101.96	138.13	35.48
Bulgaria	10557.88	5798.75	-45.08	9911.24	6947.67	-29.90
Burkina Faso	373.32	691.67	85.27	528.92	1061.54	100.70
Burundi	621.63	511.43	-17.73	583.30	705.09	20.88
Cabo Verde	21.54	37.28	73.04	35.33	57.37	62.41
Cambodia	2411.99	5215.63	116.24	2381.09	5933.78	149.20
Cameroon	579.48	1382.50	138.58	871.08	2050.57	135.40
Canada	14125.67	7327.47	-48.13	19181.66	21521.95	12.20
Central African Republic	320.94	440.64	37.30	204.99	318.16	55.21

Chad	375.39	741.01	97.39	533.25	1039.62	94.96
Chile	2100.05	1772.30	-15.61	3881.92	5761.65	48.42
China	425765.08	784390.90	84.23	511594.27	1188145.93	132.24
Colombia	4014.77	4099.64	2.11	3817.46	5752.20	50.68
Comoros	40.50	55.36	36.69	54.40	91.29	67.80
Congo	266.08	533.82	100.62	191.78	527.33	174.97
Cook Islands	5.35	5.58	4.35	6.87	10.17	48.08
Costa Rica	381.81	497.76	30.37	439.68	834.02	89.69
Croatia	4849.50	2806.26	-42.13	4562.41	3997.20	-12.39
Cuba	5268.60	4526.55	-14.08	4172.08	4952.89	18.72
Cyprus	373.45	376.04	0.69	346.37	575.23	66.07
Czechia	11646.07	4932.45	-57.65	10311.26	9743.72	-5.50
Côte d'Ivoire	824.31	1703.51	106.66	1234.56	2540.52	105.78
Democratic People's Republic of Korea	6828.46	13431.29	96.70	10032.28	16524.81	64.72
Democratic Republic of the Congo	2100.72	3618.00	72.23	2092.87	3946.18	88.55
Denmark	6836.03	1748.63	-74.42	5743.98	3993.97	-30.47
Djibouti	37.11	147.24	296.75	59.41	232.31	291.01
Dominica	10.43	9.21	-11.69	7.31	9.58	31.03
Dominican Republic	1319.95	3100.10	134.86	1105.71	2558.61	131.40
Ecuador	957.26	1253.30	30.93	1109.22	1959.22	76.63
Egypt	20222.57	41718.11	106.29	10325.69	30224.26	192.71
El Salvador	335.08	534.77	59.60	308.56	561.65	82.02
Equatorial Guinea	49.02	69.14	41.05	34.25	79.65	132.56
Eritrea	215.19	299.69	39.27	220.69	374.08	69.50
Estonia	1337.51	418.02	-68.75	1101.82	856.46	-22.27
Eswatini	52.77	78.33	48.44	53.09	73.59	38.60
Ethiopia	2092.15	1750.01	-16.35	2070.55	2984.37	44.13
Fiji	311.79	417.08	33.77	262.04	374.64	42.97
Finland	2852.79	1392.75	-51.18	3223.77	3093.18	-4.05
France	19086.43	9297.04	-51.29	30874.65	34330.74	11.19
Gabon	83.55	140.34	67.98	79.20	161.71	104.19
Gambia	87.78	164.13	86.99	126.86	209.82	65.40
Georgia	4520.77	2764.28	-38.85	3350.70	3031.92	-9.51
Germany	61420.44	24691.16	-59.80	67635.52	60454.71	-10.62
Ghana	941.98	2183.54	131.80	1123.73	3324.14	195.81
Greece	8465.91	5576.24	-34.13	8864.46	7737.66	-12.71
Greenland	28.12	19.07	-32.16	36.20	37.92	4.75
Grenada	17.23	13.68	-20.61	10.56	15.09	42.92
Guam	27.16	57.15	110.44	40.40	86.76	114.75
Guatemala	501.59	854.71	70.40	454.34	1057.05	132.65
Guinea	430.54	771.57	79.21	637.41	1079.19	69.31
Guinea-Bissau	57.20	109.84	92.03	50.51	116.30	130.23

Guyana	179.80	143.80	-20.03	89.98	103.33	14.84
Haiti	1013.50	1237.83	22.13	519.67	802.12	54.35
Honduras	446.22	1501.42	236.48	381.57	982.07	157.37
Hungary	11083.68	4847.31	-56.27	12679.11	7062.97	-44.29
Iceland	136.31	64.79	-52.47	141.67	142.89	0.86
India	158954.02	294772.91	85.45	127393.65	219874.93	72.59
Indonesia	38755.95	113460.04	192.76	57304.82	140032.91	144.36
Iran (Islamic Republic of)	10106.98	15541.28	53.77	8881.57	23621.29	165.96
Iraq	7334.89	14424.58	96.66	5042.98	12746.89	152.77
Ireland	3183.34	854.68	-73.15	2395.66	1787.67	-25.38
Israel	1865.17	800.91	-57.06	2281.67	3141.37	37.68
Italy	28923.45	12935.10	-55.28	35526.94	27763.06	-21.85
Jamaica	344.66	364.01	5.62	375.70	522.16	38.98
Japan	40813.55	28913.43	-29.16	97743.00	86405.05	-11.60
Jordan	844.98	1930.52	128.47	976.95	4857.58	397.22
Kazakhstan	6963.00	6022.60	-13.51	8255.15	9021.21	9.28
Kenya	815.98	1880.80	130.50	2073.21	3900.58	88.14
Kiribati	36.54	76.03	108.07	53.31	102.53	92.32
Kuwait	302.26	861.51	185.02	457.39	1763.89	285.64
Kyrgyzstan	1659.62	2798.06	68.60	1588.52	2625.10	65.25
Lao People's Democratic Republic	1738.00	2476.78	42.51	1222.52	2512.28	105.50
Latvia	2489.02	1117.79	-55.09	1974.51	1459.53	-26.08
Lebanon	1630.47	1816.97	11.44	1241.45	3602.79	190.21
Lesotho	157.53	365.59	132.07	160.89	282.76	75.74
Liberia	134.33	216.71	61.33	197.80	344.74	74.29
Libya	578.47	1695.74	193.14	744.62	2061.77	176.89
Lithuania	2748.71	1712.28	-37.71	2072.16	1917.80	-7.45
Luxembourg	225.03	103.20	-54.14	222.85	261.43	17.31
Madagascar	1400.45	1584.83	13.17	1587.85	2057.01	29.55
Malawi	753.06	1697.62	125.43	990.71	1956.39	97.47
Malaysia	4663.32	8678.23	86.10	5781.46	12130.35	109.81
Maldives	64.21	80.80	25.82	81.91	199.10	143.09
Mali	273.10	792.19	190.07	478.66	1463.29	205.71
Malta	196.43	98.17	-50.02	187.47	189.06	0.85
Marshall Islands	10.67	23.72	122.37	9.07	19.73	117.51
Mauritania	164.10	188.66	14.97	267.83	401.06	49.75
Mauritius	487.37	436.64	-10.41	447.44	657.31	46.90
Mexico	8811.59	13176.90	49.54	11327.93	15403.24	35.98
Micronesia (Federated States of)	47.84	67.91	41.97	42.53	62.44	46.80
Monaco	23.52	12.77	-45.70	28.61	25.11	-12.25
Mongolia	667.83	1212.26	81.52	550.31	1567.84	184.90
Montenegro	408.67	556.49	36.17	496.75	611.07	23.02
Morocco	6237.09	8432.56	35.20	4937.94	8642.77	75.03

Mozambique	960.26	2161.01	125.05	1356.49	2646.86	95.13
Myanmar	21433.77	16677.13	-22.19	17338.61	17644.48	1.76
Namibia	212.55	299.74	41.02	230.23	309.31	34.35
Nauru	6.80	7.62	12.15	5.52	5.94	7.54
Nepal	5007.47	7731.82	54.41	4296.07	6402.64	49.03
Netherlands	9258.74	3814.49	-58.80	11762.04	9761.00	-17.01
New Zealand	1753.60	963.32	-45.07	1455.19	1704.27	17.12
Nicaragua	209.60	444.77	112.20	274.91	663.00	141.17
Niger	150.51	356.26	136.70	317.64	751.40	136.56
Nigeria	3608.10	4806.55	33.22	4575.34	7774.83	69.93
Niue	1.07	0.97	-9.43	1.07	0.95	-11.46
North Macedonia	1584.97	1665.30	5.07	1867.27	2359.42	26.36
Northern Mariana Islands	9.29	19.10	105.49	15.46	26.16	69.26
Norway	3374.27	741.10	-78.04	3498.00	2274.41	-34.98
Oman	304.54	368.39	20.96	277.50	817.02	194.41
Pakistan	21538.28	39151.53	81.78	26561.85	40129.00	51.08
Palau	5.59	10.30	84.36	5.42	11.67	115.30
Palestine	586.93	950.62	61.97	344.82	1030.91	198.97
Panama	262.96	330.46	25.67	273.24	479.24	75.39
Papua New Guinea	816.56	2050.26	151.08	730.00	2026.78	177.64
Paraguay	945.72	1400.57	48.10	956.58	1642.74	71.73
Peru	1122.31	1888.79	68.29	1365.58	3979.42	191.41
Philippines	16521.65	33729.17	104.15	16303.11	37792.41	131.81
Poland	37142.22	15487.18	-58.30	30055.00	27898.05	-7.18
Portugal	4360.40	1536.02	-64.77	5208.93	3472.80	-33.33
Puerto Rico	790.66	551.50	-30.25	726.28	1029.47	41.75
Qatar	61.70	166.20	169.35	113.33	775.63	584.43
Republic of Korea	11828.68	8084.28	-31.66	29897.35	32593.12	9.02
Republic of Moldova	2349.76	2634.14	12.10	1887.96	2350.80	24.52
Romania	15719.72	9951.17	-36.70	17564.46	13419.49	-23.60
Russian Federation	107332.79	109757.90	2.26	81990.46	108539.01	32.38
Rwanda	1277.77	1376.21	7.70	1131.20	1917.15	69.48
Saint Kitts and Nevis	9.80	6.84	-30.20	5.32	7.57	42.40
Saint Lucia	22.93	21.22	-7.48	18.14	32.64	79.98
Saint Vincent and the Grenadines	13.63	17.08	25.25	10.16	18.69	83.94
Samoa	56.91	88.26	55.07	63.01	97.61	54.91
San Marino	7.45	4.45	-40.35	13.94	16.84	20.84
Sao Tome and Principe	4.23	8.73	106.33	8.63	17.92	107.65
Saudi Arabia	1981.49	7725.01	289.86	1711.73	8299.48	384.86
Senegal	610.18	875.19	43.43	867.99	1378.69	58.84
Serbia	7730.19	6092.40	-21.19	8963.44	7913.04	-11.72
Seychelles	26.60	27.11	1.91	28.89	49.93	72.84
Sierra Leone	419.05	555.86	32.65	573.70	852.06	48.52

Singapore	670.96	516.38	-23.04	1171.70	1712.17	46.13
Slovakia	4087.01	2588.78	-36.66	4113.14	4297.80	4.49
Slovenia	762.21	373.55	-50.99	1211.01	1334.57	10.20
Solomon Islands	133.84	316.89	136.77	126.93	330.49	160.36
Somalia	522.15	843.15	61.48	628.17	1176.23	87.25
South Africa	5723.66	6507.58	13.70	10300.79	10164.95	-1.32
South Sudan	503.08	547.60	8.85	561.57	679.50	21.00
Spain	16926.80	7768.00	-54.11	22807.46	22732.20	-0.33
Sri Lanka	3793.96	3643.53	-3.97	4389.33	5153.99	17.42
Sudan	5634.52	7368.83	30.78	3283.78	6469.71	97.02
Suriname	116.66	139.59	19.65	84.76	142.34	67.93
Sweden	6962.91	2718.04	-60.96	7002.86	7641.97	9.13
Switzerland	4034.39	1697.45	-57.93	3671.74	3927.39	6.96
Syrian Arab Republic	4811.21	7618.89	58.36	3365.64	5366.92	59.46
Taiwan (Province of China)	4828.97	4309.75	-10.75	11020.99	14800.82	34.30
Tajikistan	1692.22	1462.85	-13.55	1251.62	1728.76	38.12
Thailand	11198.71	15882.46	41.82	20206.45	34975.32	73.09
Timor-Leste	111.15	348.28	213.35	138.94	338.04	143.30
Togo	325.96	770.01	136.23	441.13	966.52	119.10
Tokelau	0.72	0.59	-18.90	0.59	0.59	-0.69
Tonga	25.98	31.56	21.49	30.87	37.35	20.99
Trinidad and Tobago	355.61	345.06	-2.97	254.02	393.07	54.74
Tunisia	2765.21	4855.95	75.61	2227.34	4885.28	119.33
Turkey	23900.45	23284.53	-2.58	24982.74	34877.23	39.61
Turkmenistan	1341.43	2036.14	51.79	1025.64	1974.18	92.48
Tuvalu	5.54	7.06	27.29	4.18	6.05	44.63
Uganda	840.66	1326.47	57.79	1113.74	2134.84	91.68
Ukraine	42800.53	40173.63	-6.14	38663.96	30794.96	-20.35
United Arab Emirates	226.82	701.81	209.42	428.85	3652.98	751.80
United Kingdom	60883.25	16079.41	-73.59	49706.81	30420.18	-38.80
United Republic of Tanzania	2575.54	4837.02	87.81	3376.15	7198.23	113.21
United States of America	148674.09	94561.19	-36.40	161474.71	190320.46	17.86
United States Virgin Islands	20.99	19.61	-6.60	14.70	23.55	60.20
Uruguay	1436.35	803.12	-44.09	1798.59	1450.39	-19.36
Uzbekistan	3666.39	9243.82	152.12	3403.71	10334.25	203.62
Vanuatu	47.15	99.71	111.50	35.29	79.99	126.65
Venezuela (Bolivarian Republic of)	3219.06	5373.82	66.94	2550.06	4287.01	68.11
Viet Nam	17809.96	38137.42	114.14	21240.88	52497.55	147.15
Yemen	4396.90	8623.16	96.12	2474.95	6431.42	159.86
Zambia	571.23	1211.42	112.07	712.25	1365.04	91.65
Zimbabwe	846.12	1928.27	127.89	1215.11	1977.40	62.73

Abbreviations: YLDs, years live with disability.

Supplementary Table 3: Age-standardized mortality and YLDs rates attributable to smoking-related cardiovascular diseases across 204 countries and territories in 1990 and 2021, with temporal trends spanning from 1990 to 2021(based on GBD2021 study data)

	Age-standardized mortality rate per 100,000 population				Age-standardized YLDs rate per 100,000 population			
location	1990	2021	EAPC (95%CI)	AAPC (95%CI)	1990	2021	EPAC (95%CI)	AAPC (95%CI)
Afghanistan	43.07	34.66	-0.75 (-1.05 to -0.45)	-0.69 (-0.79to-0.6)	16.05	19.42	0.81 (0.59 to 1.03)	0.64 (0.54to0.74)
Albania	69.72	53.92	-0.55 (-0.77 to -0.33)	-0.88 (-1.72to-0.03)	58.46	54.95	-0.05 (-0.09 to 0)	-0.19 (-0.24to-0.14)
Algeria	72.21	38.04	-2.19 (-2.36 to -2.02)	-2.03 (-2.13to-1.94)	44.39	35.12	-0.9 (-0.97 to -0.82)	-0.75 (-0.81to-0.69)
American Samoa	46.65	41.25	-0.47 (-0.6 to -0.35)	-0.35 (-0.52to-0.18)	55.59	47.35	-0.63 (-0.67 to -0.59)	-0.52 (-0.55to-0.49)
Andorra	22.71	8.83	-2.91 (-3.17 to -2.64)	-3.15 (-3.51to-2.79)	40.33	28.46	-1.34 (-1.42 to -1.26)	-1.13 (-1.18to-1.07)
Angola	27.05	20.18	-1.05 (-1.29 to -0.81)	-0.9 (-1.07to-0.74)	22.14	18.12	-0.56 (-0.72 to -0.4)	-0.63 (-0.68to-0.58)
Antigua and Barbuda	20.21	9.87	-2.75 (-3.02 to -2.48)	-2.31 (-3.06to-1.55)	15.63	13.67	-0.63 (-0.72 to -0.54)	-0.44 (-0.49to-0.4)
Argentina	39.22	12.88	-3.39 (-3.47 to -3.32)	-3.48 (-3.95to-3)	45.49	27.90	-1.75 (-1.81 to -1.68)	-1.57 (-1.61to-1.53)
Armenia	67.99	52.62	-1.32 (-1.49 to -1.16)	-0.84 (-1.19to-0.5)	52.72	51.74	0.06 (0.03 to 0.09)	-0.05 (-0.09to0)
Australia	33.54	5.80	-5.78 (-5.9 to -5.66)	-5.53 (-5.8to-5.26)	38.01	19.84	-2.23 (-2.28 to -2.17)	-2.09 (-2.13to-2.06)
Austria	33.16	12.45	-3.44 (-3.66 to -3.23)	-3.11 (-3.47to-2.74)	42.96	46.37	0.2 (0.04 to 0.35)	0.25 (0.19to0.3)
Azerbaijan	70.31	59.92	-0.34 (-0.68 to 0)	-0.55 (-0.81to-0.29)	40.66	42.59	0.26 (0.13 to 0.38)	0.16 (0.09to0.23)
Bahamas	20.27	11.24	-1.91 (-2.03 to -1.78)	-1.92 (-2.06to-1.78)	14.45	13.47	-0.25 (-0.29 to -0.21)	-0.23 (-0.27to-0.2)
Bahrain	77.17	26.71	-4.16 (-4.51 to -3.8)	-3.38 (-4.33to-2.43)	37.99	29.95	-0.89 (-0.98 to -0.8)	-0.76 (-0.8to-0.72)
Bangladesh	53.52	36.96	-1.04 (-1.19 to -0.89)	-1 (-1.46to-0.55)	40.37	35.70	-0.32 (-0.46 to -0.18)	-0.39 (-0.43to-0.34)
Barbados	16.46	7.53	-3.16 (-3.46 to -2.85)	-2.4 (-3.31to-1.47)	14.49	11.06	-1.14 (-1.3 to -0.97)	-0.87 (-0.92to-0.81)
Belarus	67.70	71.21	-0.28 (-0.71 to 0.14)	0.21 (-0.62to1.04)	60.25	55.34	-0.26 (-0.34 to -0.18)	-0.24 (-0.3to-0.18)

Belgium	42.11	9.53	-4.83 (-4.99 to -4.67)	-4.74 (-4.86to-4.63)	45.98	28.87	-1.52 (-1.55 to -1.48)	-1.5 (-1.58to-1.43)
Belize	19.74	10.45	-2.48 (-2.82 to -2.13)	-1.97 (-2.55to-1.39)	18.17	15.36	-0.52 (-0.57 to -0.47)	-0.54 (-0.57to-0.51)
Benin	12.48	6.66	-2.21 (-2.36 to -2.06)	-2.03 (-2.17to-1.89)	15.64	9.26	-1.96 (-2.19 to -1.74)	-1.68 (-1.74to-1.62)
Bermuda	35.49	11.38	-3.56 (-3.87 to -3.24)	-3.63 (-4.52to-2.73)	20.76	19.19	-0.33 (-0.36 to -0.29)	-0.25 (-0.28to-0.22)
Bhutan	17.90	14.67	-0.62 (-0.69 to -0.55)	-0.63 (-0.69to-0.58)	16.76	14.42	-0.57 (-0.62 to -0.52)	-0.48 (-0.5to-0.47)
Bolivia (Plurinational State of)	20.87	9.61	-2.24 (-2.58 to -1.9)	-2.47 (-2.62to-2.33)	14.26	10.85	-0.66 (-0.86 to -0.47)	-0.87 (-0.92to-0.81)
Bosnia and Herzegovina	59.40	38.81	-1.63 (-1.81 to -1.44)	-1.31 (-1.56to-1.06)	73.24	74.43	0.2 (-0.05 to 0.44)	0.06 (0.02to0.11)
Botswana	39.26	20.35	-2.38 (-2.64 to -2.12)	-2.11 (-2.38to-1.83)	40.68	31.09	-0.92 (-0.97 to -0.88)	-0.86 (-0.91to-0.81)
Brazil	55.83	16.32	-4.11 (-4.26 to -3.95)	-3.86 (-4.05to-3.67)	42.96	20.37	-2.66 (-2.78 to -2.54)	-2.37 (-2.43to-2.32)
Brunei Darussalam	55.09	19.09	-3.2 (-3.51 to -2.88)	-3.4 (-4.01to-2.79)	83.78	32.99	-3.21 (-3.39 to -3.04)	-2.97 (-3.05to-2.88)
Bulgaria	90.78	45.31	-2.91 (-3.2 to -2.62)	-2.17 (-2.46to-1.88)	82.09	60.98	-1.06 (-1.11 to -1.02)	-0.96 (-0.98to-0.93)
Burkina Faso	8.33	7.04	-0.43 (-0.59 to -0.27)	-0.53 (-0.68to-0.39)	10.49	8.97	-0.6 (-0.69 to -0.51)	-0.49 (-0.54to-0.45)
Burundi	26.15	9.65	-3.53 (-4.12 to -2.95)	-3.19 (-3.34to-3.05)	22.11	11.31	-2.08 (-2.4 to -1.75)	-2.14 (-2.2to-2.08)
Cabo Verde	9.94	8.05	-1.3 (-1.71 to -0.88)	-0.69 (-1.38to0.01)	16.96	11.13	-1.51 (-1.61 to -1.42)	-1.35 (-1.38to-1.32)
Cambodia	56.50	46.16	-0.88 (-1.01 to -0.74)	-0.66 (-0.75to-0.57)	48.22	44.02	-0.52 (-0.68 to -0.36)	-0.3 (-0.34to-0.25)
Cameroon	12.31	10.15	-0.66 (-1.21 to -0.1)	-0.64 (-0.89to-0.38)	15.89	12.27	-0.88 (-1.07 to -0.69)	-0.83 (-0.88to-0.78)
Canada	43.74	10.00	-5.05 (-5.25 to -4.85)	-4.62 (-4.79to-4.45)	59.64	33.89	-1.9 (-1.96 to -1.84)	-1.82 (-1.85to-1.78)
Central African Republic	26.20	17.37	-1.49 (-1.68 to -1.3)	-1.33 (-1.49to-1.17)	14.73	10.65	-1.12 (-1.3 to -0.94)	-1.04 (-1.09to-0.99)
Chad	13.49	12.87	-0.48 (-0.76 to -0.2)	-0.14 (-0.41to0.12)	17.32	14.57	-0.71 (-0.82 to -0.61)	-0.55 (-0.62to-0.48)
Chile	20.55	7.12	-3.3 (-3.36 to -3.24)	-3.3 (-3.37to-3.24)	35.24	24.15	-1.48 (-1.56 to -1.39)	-1.22 (-1.26to-1.19)
China	57.04	39.64	-0.96 (-1.14 to -0.78)	-1.23 (-1.44to-1.02)	56.50	55.39	0.1 (-0.01 to 0.2)	-0.1 (-0.2to0)

Colombia	22.32	7.38	-4.08 (-4.29 to -3.87)	-3.65 (-4.18to-3.11)	19.28	10.41	-2.14 (-2.25 to -2.02)	-1.96 (-2.01to-1.91)
Comoros	21.37	11.96	-2.31 (-2.59 to -2.02)	-1.91 (-2.1to-1.71)	25.22	17.05	-1.48 (-1.57 to -1.38)	-1.26 (-1.3to-1.21)
Congo	24.38	19.64	-0.86 (-1.15 to -0.56)	-0.65 (-0.88to-0.42)	15.89	15.92	0.2 (0.02 to 0.38)	0.01 (-0.03to0.04)
Cook Islands	41.01	22.50	-1.93 (-2.09 to -1.78)	-1.92 (-2to-1.84)	48.68	42.81	-0.6 (-0.67 to -0.53)	-0.43 (-0.48to-0.37)
Costa Rica	22.26	8.99	-3.12 (-3.26 to -2.98)	-3.12 (-3.27to-2.97)	23.65	15.14	-1.48 (-1.56 to -1.39)	-1.43 (-1.46to-1.39)
Côte d'Ivoire	19.77	14.34	-1.76 (-2.23 to -1.3)	-3.36 (-3.65to-3.07)	72.97	51.98	-1.39 (-1.62 to -1.16)	-1.07 (-1.12to-1.02)
Croatia	86.42	30.31	-3.18 (-3.29 to -3.07)	-2.5 (-2.77to-2.23)	40.73	26.77	-0.96 (-1.04 to -0.88)	-1.35 (-1.38to-1.31)
Cuba	52.59	23.08	-3 (-3.21 to -2.78)	-3.47 (-3.79to-3.16)	41.25	28.87	-1.58 (-1.7 to -1.46)	-1.13 (-1.18to-1.07)
Cyprus	56.24	18.82	-3.79 (-3.93 to -3.65)	-4.12 (-4.29to-3.94)	77.45	51.89	-1.33 (-1.4 to -1.26)	-1.28 (-1.3to-1.25)
Czechia	84.68	22.94	-4.23 (-4.3 to -4.15)	-1.06 (-1.21to-0.9)	22.35	16.59	-1.25 (-1.27 to -1.22)	-0.97 (-1.05to-0.88)
Democratic People's Republic of Korea	41.85	40.06	-0.24 (-0.39 to -0.09)	-0.14 (-0.17to-0.11)	54.68	48.14	-0.52 (-0.58 to -0.47)	-0.41 (-0.46to-0.36)
Democratic Republic of the Congo	12.91	8.88	-1.26 (-1.54 to -0.98)	-1.24 (-1.41to-1.07)	11.04	8.08	-0.98 (-1.13 to -0.83)	-1 (-1.03to-0.97)
Denmark	82.12	13.90	-6.16 (-6.34 to -5.98)	-5.59 (-5.77to-5.4)	74.38	37.27	-2.5 (-2.62 to -2.39)	-2.21 (-2.27to-2.15)
Djibouti	27.60	23.67	-0.55 (-0.58 to -0.52)	-0.49 (-0.59to-0.39)	34.65	29.49	-0.45 (-0.5 to -0.4)	-0.52 (-0.53to-0.5)
Dominica	17.85	10.94	-1.65 (-2.03 to -1.27)	-1.55 (-1.72to-1.39)	12.80	11.30	-0.42 (-0.57 to -0.26)	-0.42 (-0.48to-0.37)
Dominican Republic	39.16	31.27	-0.22 (-0.49 to 0.05)	-0.66 (-1.31to0)	28.74	25.29	-0.42 (-0.59 to -0.26)	-0.41 (-0.44to-0.37)
Ecuador	18.31	7.76	-2.84 (-3.24 to -2.44)	-2.85 (-3.62to-2.07)	19.51	11.66	-1.69 (-1.77 to -1.6)	-1.65 (-1.68to-1.63)
Egypt	80.51	71.96	-0.08 (-0.28 to 0.13)	-0.45 (-0.91to0.02)	32.77	41.02	0.86 (0.78 to 0.93)	0.73 (0.69to0.76)
El Salvador	10.98	8.69	-0.78 (-1.05 to -0.51)	-0.7 (-1.77to0.39)	9.73	9.26	-0.09 (-0.15 to -0.03)	-0.16 (-0.19to-0.12)
Equatorial Guinea	23.99	12.97	-2.35 (-2.81 to -1.88)	-1.91 (-2.29to-1.54)	15.19	11.62	-0.87 (-0.93 to -0.81)	-0.86 (-0.89to-0.83)

Eritrea	14.52	8.60	-1.99 (-2.18 to -1.8)	-1.7 (-1.83to-1.57)	13.05	9.16	-1.3 (-1.44 to -1.17)	-1.13 (-1.17to-1.1)
Estonia	65.86	16.17	-5.21 (-5.73 to -4.69)	-4.45 (-5.07to-3.82)	55.66	39.93	-1.17 (-1.35 to -1)	-1.05 (-1.11to-0.99)
Eswatini	20.11	14.49	-0.69 (-1.18 to -0.2)	-1.04 (-1.29to-0.79)	17.63	12.29	-1.32 (-1.41 to -1.23)	-1.16 (-1.19to-1.12)
Ethiopia	9.55	3.93	-2.84 (-3.16 to -2.52)	-2.81 (-2.92to-2.71)	8.55	5.77	-1.12 (-1.3 to -0.93)	-1.23 (-1.32to-1.15)
Fiji	74.51	49.24	-1.52 (-1.67 to -1.38)	-1.35 (-1.61to-1.1)	56.29	41.88	-1.05 (-1.13 to -0.97)	-0.95 (-0.98to-0.93)
Finland	41.07	11.25	-3.99 (-4.12 to -3.87)	-4.12 (-4.26to-3.97)	48.16	31.19	-1.27 (-1.42 to -1.12)	-1.37 (-1.45to-1.29)
France	22.93	6.61	-4.11 (-4.21 to -4.01)	-3.92 (-4.12to-3.73)	40.00	30.16	-0.91 (-0.96 to -0.86)	-0.9 (-0.93to-0.87)
Gabon	14.51	12.70	-0.46 (-0.53 to -0.4)	-0.36 (-0.47to-0.24)	13.03	12.80	-0.08 (-0.15 to -0.02)	-0.05 (-0.09to-0.02)
Gambia	24.00	16.11	-1.6 (-1.8 to - 1.41)	-1.21 (-1.56to-0.85)	28.92	17.12	-1.9 (-1.97 to -1.82)	-1.67 (-1.72to-1.63)
Georgia	72.81	47.48	-1.69 (-2.08 to -1.31)	-1.33 (-2.03to-0.62)	53.58	57.64	0.34 (0.26 to 0.41)	0.23 (0.18to0.29)
Germany	48.12	12.58	-4.43 (-4.6 to - 4.25)	-4.21 (-4.39to-4.04)	57.41	37.89	-1.34 (-1.39 to -1.29)	-1.33 (-1.39to-1.27)
Ghana	15.96	13.74	-0.09 (-0.25 to 0.06)	-0.47 (-0.64to-0.3)	15.27	16.41	0.62 (0.41 to 0.83)	0.26 (0.19to0.33)
Greece	57.32	23.59	-3.11 (-3.23 to -2.98)	-2.82 (-3.16to-2.48)	61.13	40.03	-1.53 (-1.6 to -1.46)	-1.36 (-1.42to-1.31)
Greenland	81.50	27.18	-3.63 (-3.75 to -3.51)	-3.36 (-3.57to-3.14)	88.48	51.48	-1.96 (-2.07 to -1.84)	-1.73 (-1.78to-1.67)
Grenada	25.53	11.54	-3.01 (-3.22 to -2.79)	-2.5 (-3.48to-1.51)	16.52	12.36	-1.21 (-1.3 to -1.12)	-0.95 (-1.01to-0.88)
Guam	31.28	28.04	0.1 (-0.09 to 0.29)	-0.38 (-0.79to0.02)	40.00	43.59	0.3 (0.25 to 0.35)	0.27 (0.24to0.3)
Guatemala	15.79	8.14	-2.47 (-2.78 to -2.16)	-1.95 (-2.66to-1.23)	11.96	9.19	-0.92 (-1.02 to -0.82)	-0.84 (-0.86to-0.83)
Guinea	13.26	13.72	0.28 (0.09 to 0.47)	0.11 (-0.03to0.26)	17.84	16.29	-0.3 (-0.36 to -0.23)	-0.3 (-0.33to-0.27)
Guinea-Bissau	13.52	13.23	0.33 (0.13 to 0.53)	-0.03 (-0.19to0.13)	10.40	11.37	0.62 (0.41 to 0.84)	0.31 (0.24to0.37)
Guyana	44.93	21.39	-1.98 (-2.15 to -1.81)	-2.25 (-2.74to-1.74)	20.40	14.66	-1.13 (-1.18 to -1.08)	-1.06 (-1.1to-1.03)
Haiti	29.51	16.40	-1.77 (-2 to - 1.54)	-1.85 (-1.97to-1.72)	13.69	9.15	-1.43 (-1.57 to -1.3)	-1.3 (-1.34to-1.26)
Honduras	21.85	24.75	0.71 (0.51 to 0.92)	0.42 (0.21to0.64)	17.13	14.39	-0.65 (-0.7 to -0.61)	-0.56 (-0.6to-0.53)

Hungary	77.46	26.22	-3.81 (-3.94 to -3.68)	-3.39 (-3.72to-3.05)	90.61	43.90	-2.55 (-2.63 to -2.47)	-2.3 (-2.39to-2.22)
Iceland	46.98	10.74	-4.95 (-5.05 to -4.86)	-4.7 (-4.87to-4.54)	51.45	27.19	-2.36 (-2.49 to -2.24)	-2.05 (-2.1to-2.01)
India	34.19	25.42	-0.89 (-1.01 to -0.77)	-0.92 (-1.29to-0.55)	25.06	17.74	-1.13 (-1.16 to -1.1)	-1.11 (-1.15to-1.08)
Indonesia	39.31	49.04	0.85 (0.66 to 1.03)	0.7 (0.58to0.82)	50.58	51.69	0.08 (-0.01 to 0.17)	0.07 (0.06to0.09)
Iran (Islamic Republic of)	38.12	19.84	-2.09 (-2.21 to -1.97)	-2.12 (-2.23to-2)	28.36	26.30	-0.19 (-0.25 to -0.13)	-0.24 (-0.26to-0.21)
Iraq	94.38	66.82	-1.71 (-1.89 to -1.52)	-1.06 (-1.25to-0.88)	57.89	45.72	-0.79 (-0.81 to -0.77)	-0.76 (-0.78to-0.74)
Ireland	78.85	10.53	-6.73 (-6.96 to -6.5)	-6.27 (-6.66to-5.88)	59.59	24.17	-3.38 (-3.54 to -3.22)	-2.89 (-2.96to-2.82)
Israel	39.58	6.37	-6.24 (-6.41 to -6.06)	-5.76 (-6.21to-5.32)	47.92	27.59	-1.86 (-1.96 to -1.76)	-1.76 (-1.79to-1.73)
Italy	33.08	8.48	-4.59 (-4.69 to -4.5)	-4.37 (-4.52to-4.23)	43.09	23.63	-2.05 (-2.12 to -1.98)	-1.92 (-1.99to-1.85)
Jamaica	19.52	11.69	-1.58 (-2.07 to -1.07)	-1.45 (-2.33to-0.56)	21.95	17.00	-1.12 (-1.21 to -1.02)	-0.83 (-0.86to-0.79)
Japan	24.85	8.60	-3.53 (-3.63 to -3.44)	-3.37 (-3.48to-3.26)	57.51	34.58	-1.87 (-1.95 to -1.79)	-1.64 (-1.69to-1.6)
Jordan	66.21	27.17	-3.38 (-3.6 to -3.15)	-2.79 (-3.09to-2.49)	61.57	54.53	-0.43 (-0.48 to -0.38)	-0.39 (-0.41to-0.36)
Kazakhstan	52.86	33.14	-2.51 (-3.23 to -1.79)	-1.45 (-1.92to-0.98)	58.54	44.78	-0.91 (-1.09 to -0.74)	-0.83 (-0.93to-0.73)
Kenya	10.55	8.27	-1.05 (-1.25 to -0.85)	-0.78 (-0.93to-0.64)	22.50	14.18	-1.88 (-2.03 to -1.73)	-1.49 (-1.56to-1.41)
Kiribati	90.57	93.51	-0.01 (-0.19 to 0.17)	0.11 (0.07to0.14)	117.69	112.63	-0.29 (-0.5 to -0.08)	-0.14 (-0.22to-0.06)
Kuwait	44.64	25.99	-1.67 (-2.16 to -1.17)	-1.82 (-4.46to0.89)	48.88	41.05	-0.6 (-0.68 to -0.52)	-0.56 (-0.61to-0.51)
Kyrgyzstan	56.90	61.01	0.63 (0.19 to 1.08)	0.32 (-0.07to0.72)	50.13	47.23	0.08 (-0.02 to 0.19)	-0.16 (-0.26to-0.07)
Lao People's Democratic Republic	83.81	55.77	-1.36 (-1.42 to -1.3)	-1.3 (-1.39to-1.2)	53.62	48.06	-0.31 (-0.33 to -0.3)	-0.34 (-0.36to-0.32)
Latvia	70.54	30.33	-3.33 (-3.71 to -2.95)	-2.61 (-3.89to-1.32)	57.14	45.38	-0.66 (-0.7 to -0.61)	-0.72 (-0.76to-0.68)
Lebanon	76.81	29.00	-3.25 (-3.5 to -2.99)	-3.06 (-3.31to-2.82)	52.94	60.59	0.4 (0.27 to 0.53)	0.44 (0.41to0.46)
Lesotho	19.96	35.11	2.68 (2.25 to 3.11)	1.98 (1.72to2.25)	18.70	24.86	1.17 (1.09 to 1.24)	0.93 (0.89to0.97)

Liberia	11.69	9.03	-1.16 (-1.35 to -0.97)	-0.81 (-1.09to-0.52)	15.39	11.50	-1.04 (-1.12 to -0.96)	-0.94 (-0.97to-0.91)
Libya	30.33	30.64	0.51 (0.29 to 0.72)	0.15 (-0.27to0.57)	33.77	31.03	-0.17 (-0.21 to -0.14)	-0.26 (-0.31to-0.21)
Lithuania	61.93	30.56	-2.38 (-2.7 to -2.05)	-2.27 (-2.9to-1.63)	47.35	41.66	0.53 (0.23 to 0.84)	-0.26 (-0.4to-0.13)
Luxembourg	41.71	9.48	-4.96 (-5.1 to -4.82)	-4.75 (-5.1to-4.39)	42.85	26.51	-1.92 (-2.05 to -1.79)	-1.57 (-1.62to-1.52)
Madagascar	27.66	12.75	-2.77 (-3.06 to -2.48)	-2.44 (-2.65to-2.24)	27.34	13.51	-2.45 (-2.7 to -2.21)	-2.24 (-2.32to-2.16)
Malawi	21.38	23.11	-0.04 (-0.34 to 0.25)	0.29 (0.14to0.44)	24.18	23.14	-0.21 (-0.33 to -0.1)	-0.13 (-0.16to-0.09)
Malaysia	50.64	30.94	-1.71 (-1.85 to -1.56)	-1.6 (-1.83to-1.37)	55.24	40.22	-1.05 (-1.15 to -0.94)	-1.02 (-1.04to-0.99)
Maldives	80.56	24.86	-4.25 (-4.43 to -4.07)	-3.84 (-4.08to-3.61)	83.47	45.91	-2.23 (-2.41 to -2.06)	-1.91 (-2.01to-1.81)
Mali	7.27	9.80	1.49 (1.26 to 1.72)	1.04 (0.78to1.29)	10.61	14.45	1.27 (1.17 to 1.37)	1.01 (0.96to1.06)
Malta	46.41	10.82	-4.79 (-4.93 to -4.64)	-4.68 (-5.46to-3.9)	43.54	24.44	-2.03 (-2.16 to -1.9)	-1.85 (-1.92to-1.78)
Marshall Islands	58.53	56.64	0.01 (-0.08 to 0.1)	-0.08 (-0.2to0.04)	43.00	42.46	-0.04 (-0.09 to 0.01)	-0.03 (-0.07to0.01)
Mauritania	15.74	8.42	-2.34 (-2.59 to -2.09)	-2.04 (-2.27to-1.8)	22.76	15.14	-1.44 (-1.51 to -1.38)	-1.3 (-1.34to-1.27)
Mauritius	65.32	24.45	-3.76 (-4.19 to -3.32)	-2.97 (-4.12to-1.81)	55.27	35.95	-1.59 (-1.8 to -1.38)	-1.37 (-1.46to-1.28)
Mexico	23.18	10.60	-2.91 (-3.1 to -2.71)	-2.44 (-2.64to-2.23)	24.88	11.82	-2.7 (-2.83 to -2.58)	-2.37 (-2.42to-2.32)
Micronesia (Federated States of)	91.40	78.74	-0.47 (-0.52 to -0.43)	-0.49 (-0.54to-0.43)	75.60	68.49	-0.37 (-0.41 to -0.32)	-0.32 (-0.33to-0.3)
Monaco	33.75	13.06	-3.18 (-3.28 to -3.07)	-3.01 (-3.14to-2.88)	48.38	31.89	-1.56 (-1.65 to -1.48)	-1.35 (-1.39to-1.3)
Mongolia	64.02	51.00	-0.79 (-1.14 to -0.44)	-0.82 (-1.08to-0.56)	46.95	54.50	0.66 (0.52 to 0.79)	0.49 (0.42to0.56)
Montenegro	65.72	57.96	-0.5 (-0.77 to -0.23)	-0.16 (-0.65to0.33)	76.97	64.90	-0.61 (-0.8 to -0.43)	-0.53 (-0.59to-0.47)
Morocco	43.24	24.05	-2.05 (-2.25 to -1.85)	-1.85 (-1.95to-1.74)	31.01	22.75	-1.19 (-1.3 to -1.09)	-0.98 (-1.06to-0.9)
Mozambique	16.31	18.24	1 (0.78 to 1.23)	0.39 (0.27to0.52)	19.97	19.46	0.08 (0 to 0.16)	-0.08 (-0.11to-0.05)
Myanmar	97.36	35.84	-3.53 (-3.66 to -3.41)	-3.19 (-3.27to-3.1)	70.08	33.98	-2.51 (-2.58 to -2.43)	-2.3 (-2.35to-2.26)

Namibia	39.00	25.17	-1.78 (-2.12 to -1.45)	-1.32 (-1.57to-1.07)	36.15	22.38	-1.8 (-1.91 to -1.69)	-1.54 (-1.58to-1.5)
Nauru	126.02	110.04	-0.57 (-0.91 to -0.22)	-0.44 (-0.54to-0.33)	89.61	76.82	-0.6 (-0.7 to -0.51)	-0.49 (-0.55to-0.44)
Nepal	56.93	36.12	-1.48 (-1.64 to -1.33)	-1.47 (-1.64to-1.3)	42.79	26.94	-1.78 (-1.91 to -1.64)	-1.49 (-1.54to-1.43)
Netherlands	46.43	10.25	-5.15 (-5.31 to -4.98)	-4.76 (-4.91to-4.61)	61.35	31.29	-2.29 (-2.37 to -2.21)	-2.15 (-2.2to-2.09)
New Zealand	44.74	11.23	-4.67 (-4.89 to -4.45)	-4.39 (-4.59to-4.2)	37.74	22.16	-1.94 (-2.12 to -1.75)	-1.71 (-1.74to-1.68)
Nicaragua	13.88	9.25	-0.95 (-1.15 to -0.75)	-1.38 (-1.8to-0.97)	16.14	12.64	-0.63 (-0.79 to -0.48)	-0.78 (-0.81to-0.75)
Niger	5.65	4.91	-0.44 (-0.52 to -0.36)	-0.43 (-0.62to-0.23)	8.75	7.41	-0.73 (-0.8 to -0.66)	-0.53 (-0.58to-0.48)
Nigeria	8.59	5.25	-1.78 (-2.01 to -1.55)	-1.6 (-1.78to-1.42)	9.20	6.81	-1.02 (-1.25 to -0.8)	-0.96 (-0.99to-0.94)
Niue	50.77	44.30	-0.69 (-0.79 to -0.58)	-0.46 (-0.58to-0.35)	52.48	44.72	-0.67 (-0.73 to -0.6)	-0.52 (-0.54to-0.5)
North Macedonia	86.93	57.30	-1.66 (-2.08 to -1.25)	-1.41 (-1.88to-0.94)	93.25	71.47	-0.97 (-1 to -0.93)	-0.85 (-0.89to-0.82)
Northern Mariana Islands	39.72	33.10	-0.48 (-0.66 to -0.3)	-0.58 (-0.84to-0.32)	53.99	43.58	-0.82 (-0.88 to -0.76)	-0.7 (-0.81to-0.58)
Norway	49.46	7.14	-6.7 (-6.95 to -6.45)	-6.11 (-6.52to-5.7)	57.67	26.09	-3.03 (-3.21 to -2.85)	-2.54 (-2.61to-2.48)
Oman	42.60	17.57	-2.5 (-2.63 to -2.38)	-2.78 (-3.19to-2.36)	26.32	21.60	-0.6 (-0.7 to -0.5)	-0.64 (-0.7to-0.58)
Pakistan	39.33	32.01	-0.89 (-1.16 to -0.61)	-0.67 (-0.76to-0.59)	44.80	29.12	-1.49 (-1.62 to -1.36)	-1.38 (-1.42to-1.34)
Palau	53.78	42.31	-0.77 (-0.82 to -0.71)	-0.79 (-0.99to-0.6)	48.22	45.92	-0.3 (-0.38 to -0.22)	-0.16 (-0.19to-0.13)
Palestine	73.47	40.48	-2.21 (-2.48 to -1.93)	-1.94 (-2.13to-1.75)	36.60	33.39	-0.45 (-0.5 to -0.39)	-0.3 (-0.33to-0.26)
Panama	18.36	7.40	-3.16 (-3.39 to -2.93)	-2.78 (-3.29to-2.26)	17.69	10.86	-1.82 (-1.94 to -1.7)	-1.58 (-1.64to-1.52)
Papua New Guinea	38.80	33.01	-0.58 (-0.65 to -0.52)	-0.54 (-0.75to-0.33)	31.64	29.46	-0.36 (-0.42 to -0.29)	-0.23 (-0.26to-0.19)
Paraguay	44.66	24.78	-1.82 (-2.01 to -1.63)	-1.73 (-2.03to-1.43)	41.98	27.49	-1.59 (-1.66 to -1.51)	-1.36 (-1.42to-1.3)
Peru	9.48	5.56	-2.27 (-2.77 to -1.76)	-1.62 (-2.93to-0.29)	10.67	11.51	0.01 (-0.11 to 0.13)	0.23 (0.14to0.33)
Philippines	59.60	39.54	-1.27 (-1.4 to -1.14)	-1.22 (-1.32to-1.11)	49.46	40.84	-0.67 (-0.87 to -0.46)	-0.61 (-0.66to-0.56)

Poland	86.13	21.88	-4.73 (-4.87 to -4.6)	-4.47 (-4.94to-4.01)	68.70	43.43	-1.65 (-1.7 to -1.61)	-1.48 (-1.52to-1.43)
Portugal	31.85	7.11	-5.34 (-5.63 to -5.05)	-4.83 (-5.1to-4.56)	38.94	18.80	-2.64 (-2.76 to -2.52)	-2.33 (-2.4to-2.25)
Puerto Rico	22.34	8.19	-3.73 (-3.99 to -3.48)	-3.09 (-3.51to-2.67)	20.05	17.28	-0.59 (-0.65 to -0.54)	-0.49 (-0.52to-0.46)
Qatar	52.81	14.25	-4.82 (-5.46 to -4.17)	-4.21 (-5.06to-3.36)	42.64	29.79	-1.12 (-1.24 to -1.01)	-1.13 (-1.18to-1.09)
Republic of Korea	44.96	8.89	-5.76 (-5.96 to -5.56)	-5.12 (-5.34to-4.91)	89.15	36.71	-3.28 (-3.48 to -3.09)	-2.82 (-2.94to-2.71)
Republic of Moldova	54.50	44.35	-1.1 (-1.47 to -0.72)	-0.72 (-1.95to0.52)	40.60	41.53	0.14 (0.07 to 0.22)	0.1 (0.06to0.14)
Romania	59.08	28.50	-3.27 (-3.62 to -2.92)	-2.35 (-2.85to-1.85)	62.14	43.20	-1.42 (-1.52 to -1.32)	-1.16 (-1.24to-1.08)
Russian Federation	59.98	46.84	-1.45 (-2.24 to -0.64)	-0.65 (-1.75to0.47)	44.99	48.79	0.24 (0.01 to 0.48)	0.27 (0.21to0.34)
Rwanda	49.00	25.50	-3.08 (-3.47 to -2.69)	-2.1 (-2.25to-1.95)	37.85	29.21	-0.94 (-0.97 to -0.91)	-0.83 (-0.86to-0.8)
Saint Kitts and Nevis	27.10	10.11	-3.57 (-3.79 to -3.35)	-3.04 (-3.45to-2.62)	15.52	10.01	-1.89 (-2.07 to -1.7)	-1.42 (-1.47to-1.37)
Saint Lucia	27.39	8.82	-4.17 (-4.44 to -3.89)	-3.54 (-3.96to-3.12)	20.38	13.45	-1.63 (-1.78 to -1.48)	-1.32 (-1.41to-1.23)
Saint Vincent and the Grenadines	19.43	12.15	-1.76 (-1.91 to -1.61)	-1.48 (-1.9to-1.05)	14.34	12.98	-0.46 (-0.5 to -0.42)	-0.33 (-0.37to-0.3)
Samoa	64.66	58.70	-0.41 (-0.49 to -0.33)	-0.31 (-0.36to-0.26)	66.30	61.07	-0.43 (-0.55 to -0.31)	-0.27 (-0.33to-0.2)
San Marino	20.92	5.51	-3.64 (-3.96 to -3.32)	-4.48 (-4.77to-4.19)	42.60	27.27	-1.7 (-1.81 to -1.59)	-1.44 (-1.49to-1.39)
Sao Tome and Principe	6.42	7.20	0.15 (-0.22 to 0.53)	0.39 (0.06to0.72)	12.68	12.40	-0.35 (-0.59 to -0.1)	-0.08 (-0.14to-0.02)
Saudi Arabia	30.23	27.20	-0.12 (-0.36 to 0.11)	-0.37 (-0.48to-0.26)	20.97	24.81	0.62 (0.56 to 0.69)	0.55 (0.52to0.58)
Senegal	17.63	10.58	-1.9 (-2.01 to -1.78)	-1.65 (-1.98to-1.32)	22.54	14.52	-1.59 (-1.65 to -1.53)	-1.41 (-1.45to-1.36)
Serbia	74.69	37.33	-2.74 (-3.16 to -2.31)	-2.11 (-2.42to-1.8)	74.80	55.42	-1.14 (-1.31 to -0.98)	-0.95 (-1.02to-0.88)
Seychelles	47.62	23.40	-2.27 (-2.47 to -2.08)	-2.33 (-3.09to-1.57)	51.45	40.13	-0.97 (-1.04 to -0.89)	-0.8 (-0.84to-0.75)
Sierra Leone	20.17	13.60	-1.11 (-1.29 to -0.92)	-1.28 (-1.38to-1.19)	24.68	17.70	-1.11 (-1.16 to -1.07)	-1.07 (-1.09to-1.05)
Singapore	29.48	5.91	-5.15 (-5.25 to -5.05)	-4.92 (-5.3to-4.55)	46.52	19.40	-2.94 (-3.07 to -2.8)	-2.78 (-2.84to-2.72)

Slovakia	69.23	27.33	-2.91 (-3.01 to -2.81)	-2.96 (-3.44to-2.48)	69.57	48.38	-1.08 (-1.14 to -1.02)	-1.16 (-1.19to-1.13)
Slovenia	30.95	8.49	-4.36 (-4.48 to -4.23)	-4.05 (-4.48to-3.61)	50.06	36.88	-0.74 (-0.84 to -0.64)	-0.98 (-1.01to-0.94)
Solomon Islands	91.46	82.13	-0.16 (-0.41 to 0.09)	-0.35 (-0.53to-0.17)	76.17	75.48	0.13 (0.03 to 0.22)	-0.03 (-0.06to0)
Somalia	20.17	12.34	-1.64 (-1.76 to -1.53)	-1.56 (-1.64to-1.47)	19.28	13.98	-1.11 (-1.2 to -1.01)	-1.02 (-1.08to-0.96)
South Africa	27.09	13.64	-2.45 (-2.74 to -2.15)	-2.21 (-2.95to-1.47)	46.11	19.72	-2.91 (-3.08 to -2.74)	-2.71 (-2.76to-2.66)
South Sudan	19.63	13.83	-1.38 (-1.66 to -1.1)	-1.13 (-1.28to-0.97)	20.26	14.62	-1.09 (-1.12 to -1.05)	-1.05 (-1.07to-1.02)
Spain	31.80	8.07	-4.64 (-4.77 to -4.51)	-4.32 (-4.53to-4.12)	44.73	27.92	-1.56 (-1.63 to -1.49)	-1.51 (-1.55to-1.47)
Sri Lanka	39.02	14.22	-3.07 (-3.2 to - 2.95)	-3.11 (-3.72to-2.5)	37.33	19.11	-2.45 (-2.62 to -2.28)	-2.14 (-2.22to-2.07)
Sudan	61.02	38.04	-1.74 (-1.83 to -1.65)	-1.52 (-1.56to-1.47)	31.16	27.28	-0.52 (-0.62 to -0.42)	-0.43 (-0.46to-0.4)
Suriname	44.47	21.26	-2.62 (-2.89 to -2.36)	-2.06 (-2.68to-1.44)	30.93	21.62	-1.46 (-1.61 to -1.29)	-1.16 (-1.22to-1.11)
Sweden	44.18	11.36	-4.31 (-4.44 to -4.18)	-4.31 (-4.42to-4.19)	50.47	39.88	-0.96 (-1.03 to -0.89)	-0.76 (-0.79to-0.72)
Switzerland	37.89	8.22	-4.93 (-5 to - 4.86)	-4.89 (-5.04to-4.74)	37.92	24.62	-1.45 (-1.59 to -1.32)	-1.38 (-1.42to-1.34)
Syrian Arab Republic	92.14	62.05	-1.71 (-1.87 to -1.54)	-1.25 (-1.59to-0.9)	54.50	36.70	-1.38 (-1.42 to -1.33)	-1.26 (-1.31to-1.21)
Taiwan (Province of China)	32.06	10.40	-3.62 (-3.86 to -3.39)	-3.61 (-4.21to-3.02)	65.60	37.44	-2.04 (-2.12 to -1.95)	-1.79 (-1.85to-1.73)
Tajikistan	63.06	27.28	-2.81 (-3.13 to -2.49)	-2.72 (-3.15to-2.29)	42.17	24.60	-1.78 (-1.82 to -1.74)	-1.72 (-1.75to-1.68)
Thailand	32.97	15.05	-3.11 (-3.31 to -2.92)	-2.54 (-2.87to-2.21)	51.45	33.03	-1.53 (-1.58 to -1.48)	-1.42 (-1.46to-1.38)
Timor-Leste	40.88	43.67	0.43 (0.24 to 0.62)	0.22 (0to0.45)	40.57	37.86	-0.24 (-0.26 to -0.22)	-0.23 (-0.26to-0.2)
Togo	27.10	19.34	-1.29 (-1.47 to -1.12)	-1.09 (-1.3to-0.89)	29.89	19.94	-1.35 (-1.39 to -1.32)	-1.29 (-1.37to-1.21)
Tokelau	54.12	40.17	-1.11 (-1.18 to -1.04)	-0.96 (-1to-0.91)	45.19	40.99	-0.45 (-0.51 to -0.39)	-0.31 (-0.35to-0.28)
Tonga	47.86	39.28	-0.64 (-0.78 to -0.51)	-0.65 (-0.9to-0.39)	53.21	45.45	-0.63 (-0.66 to -0.59)	-0.51 (-0.54to-0.49)
Trinidad and Tobago	42.82	17.93	-3.44 (-3.75 to -3.13)	-2.68 (-3.06to-2.3)	28.53	20.46	-1.22 (-1.29 to -1.15)	-1.06 (-1.1to-1.03)

Tunisia	62.21	38.67	-1.9 (-2.04 to -1.75)	-1.57 (-1.73to-1.4)	41.63	35.49	-0.55 (-0.58 to -0.51)	-0.51 (-0.53to-0.49)
Turkey	68.17	25.19	-3.63 (-3.92 to -3.34)	-3.18 (-3.48to-2.88)	47.12	41.79	-2.19 (-2.32 to -2.05)	-1.79 (-1.9to-1.68)
Turkmenistan	70.07	49.93	-1.86 (-2.28 to -1.43)	-1.09 (-1.95to-0.21)	54.81	53.83	-0.35 (-0.47 to -0.23)	-0.38 (-0.43to-0.32)
Tuvalu	76.61	65.08	-0.47 (-0.51 to -0.43)	-0.52 (-0.59to-0.46)	62.81	35.85	-0.13 (-0.18 to -0.08)	-0.06 (-0.08to-0.04)
Uganda	13.60	8.98	-2.42 (-2.88 to -1.95)	-1.4 (-1.65to-1.15)	15.89	12.44	-1.24 (-1.47 to -1)	-0.78 (-0.86to-0.71)
Ukraine	61.35	52.86	-1.1 (-1.75 to -0.44)	-0.49 (-1.01to0.04)	55.68	44.88	-0.66 (-0.84 to -0.48)	-0.67 (-0.74to-0.59)
United Arab Emirates	44.81	21.16	-1.47 (-1.93 to -1.01)	-2.61 (-3.69to-1.52)	42.08	38.18	-0.38 (-0.44 to -0.33)	-0.31 (-0.34to-0.28)
United Kingdom	66.75	12.02	-5.98 (-6.2 to -5.77)	-5.42 (-5.62to-5.21)	59.11	26.40	-2.96 (-3.1 to -2.81)	-2.59 (-2.66to-2.52)
United Republic of Tanzania	24.43	19.05	-1.23 (-1.39 to -1.07)	-0.8 (-0.97to-0.62)	28.20	23.92	-0.64 (-0.74 to -0.53)	-0.53 (-0.57to-0.49)
United States of America	47.07	16.57	-3.75 (-3.94 to -3.55)	-2.49 (-2.86to-2.12)	53.38	36.70	-1.51 (-1.61 to -1.42)	-0.3 (-0.33to-0.26)
United States Virgin Islands	24.65	11.34	-2.46 (-2.62 to -2.31)	-3.35 (-3.51to-3.19)	15.46	14.12	-0.36 (-0.41 to -0.31)	-1.23 (-1.28to-1.19)
Uruguay	37.85	15.52	-3.18 (-3.29 to -3.07)	-2.97 (-3.2to-2.74)	49.91	31.51	-1.81 (-1.95 to -1.67)	-1.48 (-1.55to-1.41)
Uzbekistan	31.40	35.45	0.4 (-0.17 to 0.97)	0.34 (0.01to0.68)	27.19	32.88	0.55 (0.25 to 0.86)	0.63 (0.57to0.7)
Vanuatu	67.14	50.31	-1.2 (-1.31 to -1.08)	-0.94 (-1.12to-0.77)	45.98	37.57	-0.82 (-0.95 to -0.69)	-0.65 (-0.7to-0.61)
Venezuela (Bolivarian Republic of)	33.04	17.77	-2.58 (-2.83 to -2.34)	-2.15 (-2.55to-1.76)	23.90	13.85	-1.81 (-1.86 to -1.77)	-1.74 (-1.78to-1.7)
Viet Nam	45.54	38.91	-0.46 (-0.55 to -0.36)	-0.52 (-0.57to-0.47)	50.65	48.10	-0.33 (-0.39 to -0.26)	-0.17 (-0.22to-0.13)
Yemen	86.03	60.90	-1.4 (-1.51 to -1.28)	-1.09 (-1.19to-0.99)	41.11	35.91	-0.48 (-0.52 to -0.44)	-0.43 (-0.47to-0.38)
Zambia	21.79	18.38	-0.98 (-1.16 to -0.79)	-0.54 (-0.71to-0.38)	23.57	17.46	-1.16 (-1.21 to -1.1)	-0.97 (-1to-0.93)
Zimbabwe	23.79	29.71	1.08 (0.53 to 1.63)	0.83 (0.36to1.31)	28.33	25.75	-0.27 (-0.38 to -0.17)	-0.3 (-0.33to-0.27)

Abbreviations: YLDs, years lived with disability; EAPC, estimated annual percentage change; AAPC, average annual percent change; CI, confidence intervals.

© 2025 Zhu S. et al.
